# P40 and P75 Are Singular Functional Muramidases Present in the *Lactobacillus casei /paracasei/rhamnosus* Taxon

**DOI:** 10.3389/fmicb.2019.01420

**Published:** 2019-06-26

**Authors:** Christine Bäuerl, Gulyaim Abitayeva, Sebastián Sosa-Carrillo, Ana Mencher-Beltrán, Noemí Navarro-Lleó, José M. Coll-Marqués, Manuel Zúñiga-Cabrera, Serik Shaikhin, Gaspar Pérez-Martinez

**Affiliations:** ^1^Departamento de Biotecnología, Instituto de Agroquímica y Tecnología de Alimentos (C.S.I.C.), Valencia, Spain; ^2^Department of Microbiology, L.N. Gumilyov Eurasian National University, Astana, Kazakhstan; ^3^Laboratory of Genetics and Biochemistry of Microorganisms, Republican Collection of Microorganisms at Science Committee of Ministry of Education and Science RK, Astana, Kazakhstan; ^4^Computational Biology Department, Inria, Institut Pasteur and Université Paris Diderot, Paris, France; ^5^Instituto de Ciencias de la Vid y del Vino (CSIC), Logroño, Spain

**Keywords:** P40, P75, secreted muramidases, genetic analysis, minisatellite, phylogeny, *Lactobacillus casei*, *Lactobacillus rhamnosus*

## Abstract

*Lactobacillus casei* and *Lactobacillus rhamnosus* proteins P40 and P75 belong to a large family of secreted cell wall proteins that contain a carboxy(C)-terminal CHAP or NlpC/P60 superfamily domains. In addition to their peptidoglycan hydrolases activity, proteins in this family are specific antigens of pathogens, frequently responsible of interactions with the host. *L. rhamnosus* GG and *L. casei* BL23 purified P40 and P75 proteins have antiapoptotic activity by inducing the EGF/Akt pathway. The aim of this work was to study the genetics, phylogeny and dissemination of this family of proteins in the genus *Lactobacillus* as well as their characteristics and likely function. The scrutiny of their DNA encoding sequences revealed the presence of minisatellite DNA in the P75 encoding gene of *L. casei/paracasei* strains (*cmu*B) with intraspecific indels that gave raise to four different alleles (*cmu*B1–4), which are exclusive of this species. Phylogenic analyses suggest that both proteins are present mainly in the *L. casei* and *Lactobacillus sakei* phylogenomic groups. A P40 ancestral gene was possibly present in the common ancestor of *Enterococcaceae*, *Lactobacillaceae* and *Streptococcaceae*. P75 is also present in *L. casei* and *L. sakei* groups, but its evolution is difficult to explain only by vertical transmission. Antibodies raised against the N-terminal regions of P40 and P75 improved their immunological detection in culture supernatants as they recognized almost exclusively proteins of *L. casei/paracasei/rhamnosus* strains, highlighting their structural similarity, that allowed to detect them in different fermented dairy products that contained probiotic *L. casei* strains. Purified P40 and P75 proteins showed no evident lytic activity but they complemented *L. casei* BL23 *cmu*A and *cmu*B defective mutants, respectively, thus proving that they actively participate in cell division.

## Introduction

Proteins carrying an NlpC/P60 domain constitute a large and widely distributed superfamily of bacterial proteins. However, they remain largely unexplored. An important subgroup contains the NlpC/P60 domain at the C-terminal region, and it includes phage and bacterial surface bound/secreted peptidoglycan (PGN) hydrolases, muramidases or lysins (Anantharaman and Aravind, 2003). They are also typical antigens or pathogenicity factors in bacterial pathogens (Hess et al., 1995; Park et al., 2000; Teng et al., 2003; Oechslin et al., 2013). Their enzymatic activity is determined by this conserved domain and relies on a catalytic triad formed by a cysteine that acts as a nucleophile, a catalytic histidine and a charged amino acid, frequently another histidine –but also glutamic acid, aspartic acid or asparagine-. For this reason, these domains were also called cysteine, histidine-dependent amidohydrolases/peptidases (CHAP) (Bateman and Rawlings, 2003; Aramini et al., 2008; Sanz-Gaitero et al., 2014). In Firmicutes, cell wall maintenance and reshaping during cell division involves a wide variety of enzymes (Vermassen et al., 2019); among them, CHAP-containing proteins have been associated to cell wall degradation and cell division (Layec et al., 2008). NlpC/P60 C-terminus domain proteins are highly represented in most bacterial genomes and it is very frequent to find between three to five genes encoding CHAP-containing proteins (see below). Their amino(N)-terminal regions have been related to substrate recognition, as in the case of domains LysM (Visweswaran et al., 2014) and SH3 (Gründling and Schneewind, 2006), however, there are exceptions like the N-terminal COG3883 domain of *Bacillus subtilis* CwlO PGN hydrolase (Yamaguchi et al., 2004). Cse, a PGN hydrolase from *Streptococcus thermophilus*, has a LysM N-terminal domain and it constitutes an interesting example of the complex events driving the likely evolution of these proteins. In this protein, there is a central peptide sequence (Var-Cse) encoded by consecutive tandem nucleotide repeats (TR) that are highly variable within the species. Furthermore, this Var-Cse region is necessary for normal cell division (Borges et al., 2006).

Cysteine, histidine-dependent amidohydrolases/peptidases-containing proteins are also involved in bacteria-host interactions. In *Lactobacillus rhamnosus* GG, P40 and P75 were found to inhibit epithelial cell apoptosis and to promote cell growth (Yan et al., 2007). Their homologous counterparts in *L. casei* also displayed pro-proliferative and antiapoptotic activity that was demonstrated *in vitro* and *in vivo* (Wang et al., 2014, 2016; Shen et al., 2018). These proteins induced the phosphorylation of the epidermal growth factor receptor (EGFR) and other intermediates in the EGFR/Akt signal transduction pathway (Bäuerl et al., 2010; Yan et al., 2011, 2013; Yan and Polk, 2012). In addition, it has been proposed that the functional activity of some PGN hydrolases may be explained by their ability to hydrolyse PGN ligands that induce NOD2 signal transduction pathways, thus subverting host innate immune response (Humann and Lenz, 2009). Furthermore, P40 biological function has been extended to the induction of IgA synthesis (Wang et al., 2016).

Until present, research on P40 and P75 has shown that they are secreted cell wall muramidases encoded by *cmu*A and *cmu*B in *L. casei* and *msp*B and *msp*A in *L. rhamnosus* GG (LGG) and have muramidase activity. More specifically LGG P75 has γ-D-glutamyl-L-lysyl-endopeptidase activity. The observation of knock out mutants indicated that these proteins are possibly required for the normal conformation of the cell wall or separation of bacterial daughter cells in both, *L. casei* and *L. rhamnosus* (Bäuerl et al., 2010; Regulski et al., 2012). P40 and P75 might share high similarity within *L. casei/paracasei* species, as anti-P40 antibodies recognized P40 and P75 in a large number of strains (Bäuerl et al., 2010), however, no further characterization and comparative studies have been carried out. The purpose of this work was to study the structure, physiological function, phylogenetic and genetic features in these biologically interesting proteins, in order to highlight links among strains in the *L. casei/paracasei/rhamnosus* taxon and to determine if they are exclusively present in this bacterial group.

Taxonomy remark: In this work, we respected the existing species name in the annotated sequence databases. *L. casei* BL23 and *L. rhamnosus* GG belong to the *L. casei/paracasei/rhamnosus* group, recently included in the *L. casei* phylogenomic group (Salvetti et al., 2018), a group that has some taxonomical controversy regarding *L. casei* and *L. paracasei* strains. *L. casei* BL23 is taxonomically different to the type strain *L. casei* ATCC 393^T^ (Acedo Felix and Perez Martinez, 2003) and more similar to *L. paracasei*, as well as a very large number of strains that are still designated as *L. casei*. Consequently and due to the low number of strains homologous to the standing type strain (ATCC 393^T^), *L. casei* will be equivalent to *L. paracasei* with the exception of *L. casei* ATCC 393^T^.

## Materials and Methods

### Bacterial Strains and Culture Conditions

*Lactobacillus* strains were grown on MRS medium (DIFCO) at 37°C, except *L. sakei, L. pentosus* and *L. curvatus* strains that were grown at 30°C. All lactobacilli were stored at -80°C in 15% glycerol in the laboratory’s collection and their respective origins are listed in Table 1. *Listeria monocytogenes* BL1001 (CECT 932) and *Staphylococcus aureus* BL102 (CECT 86) were grown on Brain Heart Infusion (Conda-Pronadisa) and *Enterococcus faecalis* BL141 (laboratory isolate) was cultured in Bacto^TM^ Todd Hewitt broth (Becton Dickinson) static at 37°C. The cloning hosts were *Escherichia coli* DH5α and DH10B and pQE80e derived plasmids were introduced into *E. coli* BL21(DE3)-[pLysS] for protein expression and purification. They were grown in LB medium at 37°C under agitation. Recombinant plasmids in *E. coli* were selected with ampicillin at 100 μg/ml and chloramphenicol at 20 μg/ml. Solid medium was prepared by adding 1.8% (w/v) agar. Strains were identified by PCR amplification and 16S rDNA sequencing using standard primers 27f and 1493r (Table 2) at the Genomics Service of the University of Valencia.

**Table 1:** List of *Lactobacillus* strains used in this study.

Collection number	Species	Collection number or source (Origin)
BL6	*L. casei*	ATCC393^T^ (Dairy product)
BL23	*L. casei*	[Bruce Chassy, U. Urbana (Illinois, United States)]
BL28	*L. casei*	64H (C-.A. Alpert, U. Ösnabrück, Germany)
BL32	*L. casei*	CECT 4040 (Majorero Cheese)
BL81	*L. casei*	Commercial Dairy product^∗^
BL82	*L. casei*	CECT 277 (Sour milk)
BL83	*L. casei*	CECT 4043 (Majorero Cheese)
BL86	*L. casei*	CECT 4045 (Majorero Cheese)
BL87	*L. casei*	ATCC 11578 (Oral Cavity)
BL90	*L. casei*	ATCC 334 (Emmental Cheese)
BL91	*L. casei*	ATCC 4646 (Dental Caries)
BL101	*L. casei*	Commercial Dairy product^∗^
BL193	*L. casei*	Commercial Dairy product^∗^
BL199	*L. casei*	CERELA 87 (Infant feces)
BL201	*E. faecalis*	Laboratory isolate; adult feces
BL202	*E. faecium*	Laboratory isolate; adult feces
BL203	*L. casei*	Laboratory isolate; adult feces
BL205	*L. casei* subsp. *pseudoplantarum*	Nordic fermented product^∗^
BL206	*L. casei*	Nordic fermented product^∗^
BL212	*L. casei*	ADNOX 95 (PROIMI-CERELA)
BL216	*L. casei*	CECT 5289 (Unknown)^∗∗^
BL227	*L. casei*	Commercial Dairy product^∗^
BL229	*L. casei*	Commercial Dairy product^∗^
BL312	*L. paracasei*	Commercial Dairy product^∗^
BL358	*L. casei*	Commercial Dairy product^∗^
BL1	*L. rhamnosus*	CECT 278^T^; ATCC 7469^T^
BL2	*L. rhamnosus*	CECT 276; NCIB 8651
BL3	*L. rhamnosus*	CECT 275; NCIB 8963
BL102	*L. rhamnosus*	Laboratory isolate
BL103	*L. rhamnosus*	Laboratory isolate (MRS contaminant)
BL327	*L. rhamnosus*	CECT 288; ATCC 11979(9595) (Unknown)
BL367	*L. rhamnosus*	Commercial Dairy product^∗^
BL376	*L. rhamnosus*	Commercial Dairy product^∗^
BL377	*L. rhamnosus*	ATCC 53103 (Human Feces)
BL378	*L. rhamnosus*	Commercial Dairy product^∗^
BL305	*L. casei*	(cmuA^−^) (Bäuerl et al., 2010)
BL306	*L. casei*	(cmuB^−^) (Bäuerl et al., 2010)
BL5	*L. acidophilus*	CECT 289 (Unknown)^∗∗^
BL7	*L. brevis*	CECT 216 (Beer)
BL8	*L. plantarum*	CECT 748 (Pickled cabbage)
BL13	*L. sakei*	CECT 906 (Starter of sake, moto)
BL14	*L. curvatus*	CECT 904 (Milk)
BL15	*L. alimentarius*	CECT 570^T^ (Unknown)^∗∗^
BL26	*L. plantarum*	CECT 221 (Grass silage)
BL33	*L. delbrueckii* subsp. *bulgaricus*	CECT 4005^T^ (Bulgarian Yogurt)
BL34	*L. fermentum*	CECT 4007^T^ (Fermented Beet)
BL35	*L. pentosus*	CECT 4023^T^ (Unknown)^∗∗^
BL36	*L. brevis*	CECT 4121^T^ (Human feces)
BL47	*L. pentosus*	MD353 (University of Amsterdam)
BL73	*L. acidophilus*	CNRZ 55 (INRA Collection)
BL74	*L. acidophilus*	CNRZ 216 (INRA Collection)
BL75	*L. acidophilus*	CNRZ 217 (INRA Collection)
BL76	*L. murinus*	CNRZ 220 (INRA Collection)
BL80	*L. sakei*	23K (INRA Collection, Meat product)
BL84	*L. brevis*	Laboratory Isolate (MRS contaminant)
BL89	*L. delbrueckii subsp. lactis*	DSM 7290 (Unknown)^∗∗^
BL95	*L. zeae*	ATCC15820 (Corn Step Liquor)
BL130	*L. coryniformis* subsp. *coriniformis*	CECT 982^T^ (Silage)
BL131	*L. coryniformis* subsp. *torquens*	CECT 4129^T^ (Air of cowshed (dairy barn))
BL132	*L. plantarum*	CECT 748^T^ (Pickled cabbage)
BL134	*L. paracasei* subsp. *tolerans*	CECT 4175^T^ (Pasteurized Milk)
BL135	*L. agilis*	CECT 4131^T^ (Municipal sewage)
BL156	*L. farciminis*	CECT 571^T^ (Sausage)
BL157	*L. ruminis*	CECT 4061 (Unknown)^∗∗^
BL158	*L. salivarius*	CECT 4063^T^ (Saliva)
BL159	*L. salivarius*	CECT 4062 (Saliva)
BL160	*L. delbrueckii* subsp. *bulgaricus*	Commercial Dairy product^∗^
BL166	*L. plantarum*	WCFS1 (Univ. Wageningen)
BL202	*L. acidophilus*	Laboratory isolate; feces
BL207	*L. helveticus*	Commercial Dairy product^∗^
BL 221	*L. crispatus*	M247- Ma_ Luisa Callegari (I. Microbiologia Piacenza)
BL 223	*L. gasseri*	4B2- Ma_ Luisa Callegari (I. Microbiologia Piacenza)
BL228	*L. acidophilus*	Commercial probiotic^∗^
BL231	*L. acidophilus*	Commercial probiotic^∗^
BL277	*L. gasseri*	DSMZ 20243^T^ (Human)
BL278	*L. crispatus*	DSMZ 20584^T^ (Eye)
BL279	*L. acidophilus*	CECT 4529 (Unknown)^∗∗^
BL280	*L. acidophilus*	CECT 4179 (White rat feces)
BL281	*L. johnsonii*	CECT 289 (Unknown)^∗∗^
BL287	*L. johnsonii*	DSMZ 10533^T^ (Human Blood)
BL288	*L. intestinalis*	DSMZ 6629^T^ (Rat intestine)

*Symbols in the table: (^T^) Type Strain; (^∗^) Commercial Brands are avoided in this work; (^∗∗^) Strains with undefined origin or not found in current culture collections; CECT, Colección Española de Cultivos Tipo (Spain); ATCC, American Type Culture Collection (United States); DSMZ, Deutsche Sammlung von Mikroorganismen und Zellkulturen GmbH (Germany); CERELA, Centro de Referencia para Lactobacilos (Argentina).*

**Table 2:** List of Oligonucleotides used in this study and their purposes.

Name	Oligonucleotides	Purpose	References
27f	AGAGTTTGATCMTGGCTCAG	16S rDNA sequencing	Lane, 1991
1493r	TACGGYTACCTTGTTACGACTT	16S rDNA sequencing	Lane, 1991
LcaP40N-for	GTTGGATCCGATACAAGCGACAG (BamHI)	Cloning	Bäuerl et al., 2010
LcaP40N-rev	GTAGGGCCCTTATTGCTTATTCAAAGC (SmaI)	Cloning	This work
LcaP40C-rev	GTAGGGCCCTTACCGGTGGATATAA (SmaI)	Cloning	This work
LcaP75N-for	GTTAGATCTTCAACGGGGACA (Bgl II)	Cloning	Bäuerl et al., 2010
LcaP75N-rev	TAAGGGCCCTTATTTCCCAATTTGC (SmaI)	Cloning	This work
LcaP75C-rev	TAAGGGCCCTTATAGTGAAGGACG (SmaI)	Cloning	This work
P40carh-f1	CCTTGGGGTCA**R**TGCAC**M**TGGTACG (BL23 genome position 22495-22523)	PCR detection	This work
P40carh-r	CCGGTTGGTGAGCTGAAGCCC (BL23 genome position 22296-22275)	PCR detection	This work
P75carh-f	GACCACCGTATGGAATAGTCC (BL23 genome position 277183-277204)	PCR detection	This work
P75carh-r	GG**R**ATCCACTGATTGTCGCC (BL23 genome position 277321-277301)	PCR detection	This work

*Underlined sequences in cloning primers indicate the restriction enzyme site in brackets.*

### Cloning, Expression and Purification of P40 and P75

DNA fragments were amplified from *L. casei* BL23 chromosomal DNA with proofreading Expand blend (Expand High Fidelity PCR System, Roche, Mannheim, Germany). Specially designed primers were used to amplify the mature protein encoding sequences of P40 (LcasP40N-for and LcasP40C-rev) and P75 (LcasP75N-for and LcasP75C-rev), as well as the NH-terminal domains (N-domains) of P40 (LcasP40N-for and LcasP40N-rev) and P75 (LcasP75N-for and LcasP75N-rev), respectively. Primers had additional restriction sites to facilitate subsequent cloning. Sequence details are described in Table 2. PCR amplification conditions were as follows: 94°C, 3 min; 30 cycles of 94°C, 30 s; 50–55°C, 30 s; 72°C 1.5 min; 72°C, 7 min for final extension. Amplicons were digested with *Bam*HI and *Sma*I, ligated to pQE80e carrying the RGS-His epitope encoding region and transformed in *E. coli* DH10B in order to check inserts. Fragments with the correct DNA sequence were subcloned in *E. coli* BL21(BE3)-[pLysS] to induce expression with IPTG (see below). Restriction endonucleases and ligase were purchased from Gibco BRL (Invitrogen Corp., Carslab, CA, United States) and used as recommended by the manufacturer. Nucleic acid manipulation and general cloning procedures were performed according to laboratory manuals (Sambrook and Russell, 2001).

For protein purification, recombinant bacteria were grown in 500 ml of LB supplemented with 100 μg/ml ampicillin and 20 μg/ml chloramphenicol at 37°C. When bacterial cells reached an OD600 nm of 0.4, 1 mM IPTG was added and growth continued for 3 h. The bacterial pellets were recovered by centrifugation and resuspended in 30 ml of Binding Buffer A-His (50 mM Tris–Cl pH 7.5, 100 mM NaCl, 50 mM Na_2_SO_4_) containing 0.5 mM PMSF and 0.5 mg/ml lysozyme. Bacteria were lysed by sonication, and cell debris was removed by centrifugation at 10000 × *g* for 5 min at 4°C. The resulting supernatant was filtered (pore-size 0.45 μm) and loaded onto a HisTrap^TM^ FF Crude Column (GE Healthcare Bio-Sciences AB, Uppsala, Sweden) using an ÅktaPrime^TM^ Plus chromatography system (GE Healthcare Bio-Sciences AB, Uppsala, Sweden), and His-tagged proteins were separated according to the instructions of the manufacturer. Briefly, after washing of the column with 10 volumes of Buffer A-His and 10 volumes of Buffer A-His supplemented with 40 mM imidazole, the recombinant proteins were eluted using a 20 ml linear gradient from 40 to 500 mM imidazole in Buffer A-His (Buffer B). A constant flow rate of 1 ml/min was maintained in all the purification steps (binding-washing-elution).

Protein concentration was determined using the Bio-Rad Protein Assay Dye Reagent Concentrate (Bio-Rad, CA, United States) based on the Bradford method. All purified proteins were buffer-exchanged to Tris 50 mM pH 8.0, NaCl 100 mM, 1 mM EDTA and glycerol (15% v/v) was added before storage at -80°C.

### Detection of P40 and P75 by PCR

DNA isolation of lactobacilli was performed using the REAL-pure SSS DNA^TM^ extraction kit (Durviz, Spain) according to the manufacturer’s instructions with some modifications. An aliquot of 1.5 ml of cell culture was centrifuged, the pellet was resuspended in 150 μl of RBC buffer (REAL-pure SSS DNA^TM^ extraction kit), lysozyme (15 μl of a 20 mg/ml solution) and 15 U of mutanolysin were added and the cell suspension was incubated for 30 min at 37°C. Cells were centrifuged at 11000 × *g* for 1 min, the supernatant was removed and the pellet was vortexed to resuspend in 10–20 μl of residual liquid. Then, 300 μl of lysis solution and 10 μl RNAse (10 mg/ml) were added. The remaining steps of the extraction were followed according to the instructions of the manufacturer. Quantitation of DNA was carried out in a Nanodrop^TM^ spectrophotometer (Thermo Fisher Scientific, Waltham, MA, United States). Primers used for the detection tests of P40 (P40_casrham_for1, P40_casrham_rev) and P75 (P75_casrham_for, P75_casrham_rev) were developed as described in the section “Results” (Table 2). PCR reactions contained 70 ng of total bacterial DNA as a template and amplification conditions were 95°C, 4 min; 30 cycles at 95°C, 30 s, 59°C (P40) -52°C (P75), 30 s; 72°C, 30 s; and a final extension of 72°C, 5 min.

### Antagonistic/Lytic Activity of P40 and P75

Target bacterial strains (*L. casei* BL23, *L. monocytogenes* BL1001, *E. faecalis* BL143 and *S. aureus* BS102) were grown to OD_550_ 0.4–0.6. Aliquots of 1 ml of these cultures were centrifuged, washed twice with 50 mM Tris Buffer pH 7.5, MgCl_2_ 2.5 mM and adjusted to an OD_550_ 0.1 in sterile peptone (10 g/l peptone, pH 6.3). Then, 0.9 ml of the cell suspensions were transferred to Eppendorf tubes and 100 μl of a concentrated solution of P40 and P75, individually or mixed, was added to obtain a final concentration of 50 μg/ml of each of them. Tubes were incubated at 37°C for 0 to 2 h. To test cell lysis, 55 μl of 1.0% Triton x100 was added to 445 μl of the samples taken at different times, mixed by inversion and kept on ice. When all samples were collected, OD_550_ of non-treated cell suspensions was measured against a blank of peptone, and that of Triton x100-treated samples against a blank with Triton x100.

### Isolation of Antibodies and Immunological Detection

Rabbit polyclonal antibodies were obtained by standard procedures (Leenaars and Hendriksen, 2005). Briefly, 0.5 to 1.0 mg of purified N-domains of P40 and P75 were dissolved in 0.5 ml of phosphate buffered saline (PBS) and filtered through 0.22 μm pore size membranes. Before injections, they were mixed with 0.5 ml of Complete Freund’s Adjuvant for the first injection (incomplete Freund’s Adjuvant in subsequent injections) and the mixes were administered subcutaneously in four to six sites on the back of the animals. Injections were repeated at 21 days intervals and antibody titer was tested after the second injection (ear bleeding) to decide if another boost was needed. Immunization experiments in rabbits had the approval of the Ethical Committee of University of Valencia, where the experiments were carried out, and the corresponding authorization of the Government of the Comunitat Valenciana (2014/VSC/PEA/00197). Presence of P40 and P75 was analyzed both by Dot-Blot and Western-Blot as previously described (Bäuerl et al., 2010). Briefly, protein extracts or supernatants were analyzed by SDS-PAGE and electro-transferred to a Hybond nitrocellulose membrane (GE Healthcare Life Sciences, Uppsala, Sweden). For Dot-blot analysis, 2 μl of bacterial cell extract or cell culture supernatant were manually spotted on the nitrocellulose membrane. Then the membranes were blocked in 5 % non-fat dry milk solution and incubated either with rabbit polyclonal anti-P40N or anti-P75N (1:5000 in 5% non-fat dry milk) or when indicated with rabbit polyclonal anti-P40T and anti-P75T (Yan et al., 2007) (1:10000 in 5% non-fat dry milk). After incubation with HRP-conjugated goat anti-rabbit antibody (GE-Healthcare), detection was performed using the chemiluminescent ECL kit (GE Healthcare). Light emission from the blots was detected and quantified with a LAS-1000 image analysis system (Fuji, Tokyo, Japan).

Detection of P40 and P75 in liquid dairy commercial products was assayed using cleared fractions (whey) obtained as follows: 1 ml of each product was mixed with 200 μl 2M Tris pH 7.0 and 200 μl of 50 mM EDTA (pH 8) and centrifuged in Eppendorf tubes at 11000 × *g* for 5 min. Then, aliquots of the supernatant (whey fractions) were directly mixed with loading buffer and subjected to PAGE. Transference to nitrocellulose membrane and Western detection were carried out as described above.

### Microscopic and Immunohistochemistry Techniques

The cellular distribution of P40 and P75 in *L. casei* BL23 and *L. rhamnosus* GG was first determined by microscopic epifluorescence observation. To this purpose, bacterial suspensions were fixed and treated with rabbit anti-P40N and anti-P75N polyclonal antibodies. Subsequently, the preparations were treated with fluorescein-coupled goat anti-rabbit antibodies following a standard protocol. Briefly, bacteria were grown overnight, and then diluted 1:100 in MRS and incubated for 1 h. Bacteria were fixed by placing 5 μl of the bacterial suspension on a slide, where they were mixed with 10 μl of 4% of para-formaldehyde (PFA), air dried and then gently heated. Bacteria were subsequently covered with 50 μl blocking solution (4% BSA in PBS) for 1 h at room temperature (RT). The liquid was removed gently with a pipette and bacteria were covered with 50 μl of the primary antibody solution (anti-P40N and anti-P75N) diluted 1:400 in PBS supplemented with 1% BSA and incubated for 1 h at RT. Then bacteria were washed six times with 50 μl PBS, covered with 50 μl diluted (1:500) Alexa Fluor 488 cross-absorbed goat anti-rabbit secondary antibody (Invitrogen, United States) solution in 1% BSA/PBS and incubated for 1 h at RT. Cells were washed 6 times with 50 μl PBS in the dark and fixed again with 50 μl 4% PFA for 10 min at RT in the dark. Immunolabelled bacteria were dried and covered with a drop (5 μl) of mounting medium (PBS/glycerol 1:1) before lying on the cover slide. Samples could be stored at 4°C in the dark for several weeks. Epifluorescence digital images were acquired with an Eclipse 90i Nikon microscope (Nikon Corporation, Japan) at 1000-fold magnification, equipped with a digital camera (Nikon DS-5Mc). Bright field and phase contrast digital images were also acquired with the same Eclipse 90i Nikon microscope (Nikon Corporation, Japan) and camera (Nikon DS-5Mc).

For immunohistochemistry in transmission electron microscopy, bacterial suspensions in PBS were included in 1% low-gelling temperature agarose and fixed overnight with Karnovsky fixative (2.5% paraformaldehyde, 0.5% glutaraldehyde in 0.2M cacodylate buffer pH 7.4). Samples were subsequently washed three times with PBS, post-fixed with 2% osmium tetroxide for 2 h, washed and dehydrated in ascending ethanol concentrations from 30 to 90% and embedded in LR-White resin. After polymerization of the resin, ultrathin sections were obtained with an UC6 Ultramicrotome (Leica Microsystems, Germany) with a diamond knife (Diatome, Switzerland), and placed in nickel grids. Grids were incubated for 3 h with anti-P40N and anti-P75N antibodies diluted 1:50 in PBS for 2 h. After washing in Tris 50 mM pH 8.0 containing 1% gelatin and 0.5% Tween-20 for 10 min, grids were incubated for 1 h with a 10 nm gold conjugate goat anti-rabbit antibody diluted in Tris 50 mM pH 8.0 with 1% BSA and 0.5% Tween-20 for 2 h. Subsequently, grids were washed with PBS, fixed with 2.5% glutaraldehyde in PBS for 2 min and washed in distilled water. Finally, samples were stained with 2% uranyl acetate and lead citrate for 25 and 12 min, respectively. Samples were examined in a transmission electron microscope Jeol JEM-1010 (80 kV) with a AMT RX80 (8 Mpx) digital camera.

### Sequence Analysis

Sequences of P40 and P75 homologs were retrieved from the microbial genome repository at the NCBI by PSI-BLAST (Altschul et al., 1997) using as query sequences the *L. casei* BL23 P40 (Acc. No. CAQ65155) and P75 (Acc. No. CAQ65403) protein sequences. Domain analysis was performed using the tools implemented at the NCBI Blast site^1^. The resulting datasets were aligned with M-Coffee (Notredame et al., 2000) at the T-Coffee server^2^ with default settings. Positions of uncertain homology and gaps were removed using GBLOCKS (Castresana, 2000) at the Gblocks server^3^ allowing smaller final blocks and less strict flanking positions. Redundant sequences were removed by using the EMBOSS suite Skipredundant tool (Rice et al., 2000) with a percentage sequence identity redundancy threshold of 98%. The best-fit models of amino acid substitution were selected by using the tool implemented in MEGA (ver. 7.0.18) (Kumar et al., 2016). The models selected were used to obtain maximum likelihood trees using PhyML ver. 3.0 (Guindon and Gascuel, 2003) at the PhyML server^4^. Bootstrap support values were obtained from 1000 pseudorandom replicates.

## Results

### Features of *L. casei* BL23 P40 and P75 Encoding Sequences

**FIGURE 1:**
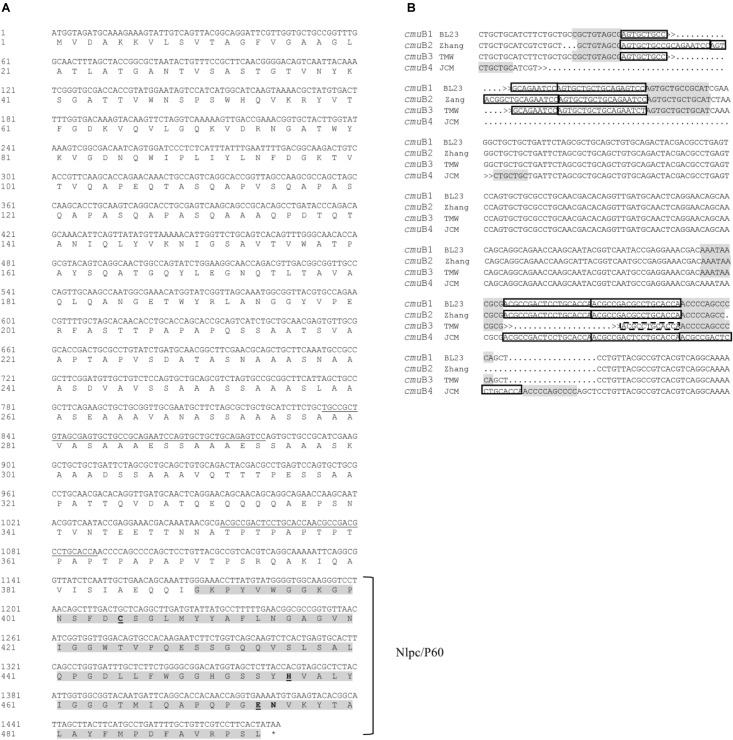
Features of *L. casei* BL23 *cmu*B, P75 encoding sequence. **(A)** The translated sequence where the NlpC/P60 domain peptide sequence is enhanced and Tandem Repeat (TR) DNA (minisatellite) are underlined in the nucleotide strand. Putative active triad amino acid residues C-H-E tentatively deduced from proposed consensus are marked in bold and underlined (Aramini et al., 2008; Sanz-Gaitero et al., 2014). **(B)**The TR sequences found in homologous genes from *L. casei* BL23 and *L. paracasei* strains that constitute four alleles. TR is framed in boxes and conserved flanking regions are shadowed in gray. Names of the alleles and representative strains are: *cmu*B1 BL23 (*L. casei* BL23)*, cmu*B2 Zhang (*L. paracasei* Zhang and identical sequences from *L. casei* LOCK919, *L. paracasei* ZFM54 and *L. paracasei* NRRL B), *cmu*B3 TMW (*L. paracasei* TMW 1.1434) and *cmu*B4 JCM (*L. paracasei* JCM 8130). GenBank references and nucleotide sequences in FASTA format can be found in Supplementary Table S2.

P40 and P75 proteins belong to a family of proteins carrying a C-terminal CHAP (NlpC/P60 superfamily) domain. At the N-terminal region, P40 has abundant positively charged amino acids included in a CwlO (COG3883) domain of the cl25603 superfamily characteristic of PGN hydrolases. Between both domains there is a serine, alanine, threonine rich spacer region with undefined similarity (Supplementary Figures S1A,B). Domains search of P75 also rendered a C-terminal CHAP region, but the N-terminus is rich in polar amino acids without known conserved domains. This protein also has a middle region with abundant serine and alanine residues and, in addition, threonine and proline rich areas (Figure 1A). Many of the protein sequences analyzed below show variable intermediate regions next to the CHAP domain. Tandem repeat (TR) search in the genes encoding P40 and P75 in *L. casei* BL23 (*cmu*A, *cmu*B) and *L. rhamnosus* GG (*msp*B, *msp*A) resulted in the localization of two consecutive 18 nt repeats or minisatellite DNA regions (AGTGCTGCCGCAGAATCC and ACGCCGACTCCTGCACCA) in the middle region of *L. casei* BL23 *cmu*B. These TR are not present in *L. rhamnosus msp*A (Supplementary Figure S2), neither in *L. zeae* (AZCT01000007.1:13555-14967) or *L. casei* subsp *casei* ATCC 393 (AP012544.1:225479-226891) homologous genes. These TR, or microsatellites, were found in a large number of homologous genes in *L. paracasei* strains, including *L. casei* ATCC 334 and BL23. The vast majority of strains included in the *L. casei/paracasei* group had identical TR structure as BL23 with a few interesting exceptions. Three instead of two copies of the first TR were found in the strains *L. casei* Zhang, *L. casei* LOCK919, *L. paracasei* ZFM54 and *L. paracasei* NRRL B, and a total deletion in *L. paracasei* JCM 8130. Two sequence divergences for the downstream second microsatellite were found showing a total deletion in *L. paracasei* TMW 1.1434 and an additional third copy in *L. paracasei* JCM 8130 (Figure 1B and Supplementary Table S3). This variability in the intermediate region of P75 in *L. casei/paracasei* conformed four alleles, *cmu*B1, *cmu*B2, *cmu*B3, and *cmu*B4 (Figure 1B). These genetic variations did not affect the phylogeny built with protein sequences shown below.

### P40 and P75 Phylogeny

As in other bacterial groups, *Lactobacillus* and closely related bacteria host several proteins with a conserved C-terminal NlpC/P60 domain, so that analyzing the annotated genomes of representative strains of *Lactobacillus* and *Lactococcus* four sequences were found in *L. casei* BL23, three in *L. rhamnosus* GG, three in *Lactococcus lactis* subsp. *cremoris* MG1363, five in *L. plantarum* WCFS1, five in *Lactobacillus delbrueckii* subsp. *bulgaricus* ATCC BAA-365, three in *Lactobacillus brevis* ATCC BAA-365 and just one in *Lactobacillus gasseri* ATCC 33323 (Supplementary Table S1).

Frequently, the conserved domains of the proteins are selected to obtain sound phylogenetic relationships as shown by other studies already performed with proteins of this family (Anantharaman and Aravind, 2003; Bateman and Rawlings, 2003; Layec et al., 2008). However, the NlpC/P60 superfamily domain covers a small part of the protein at the C-terminus, for which important information could be missed. This is particularly important if we try to find the most similar protein sequences -with muramidase activity-, but also aim to investigate other biological functions.

**FIGURE 2:**
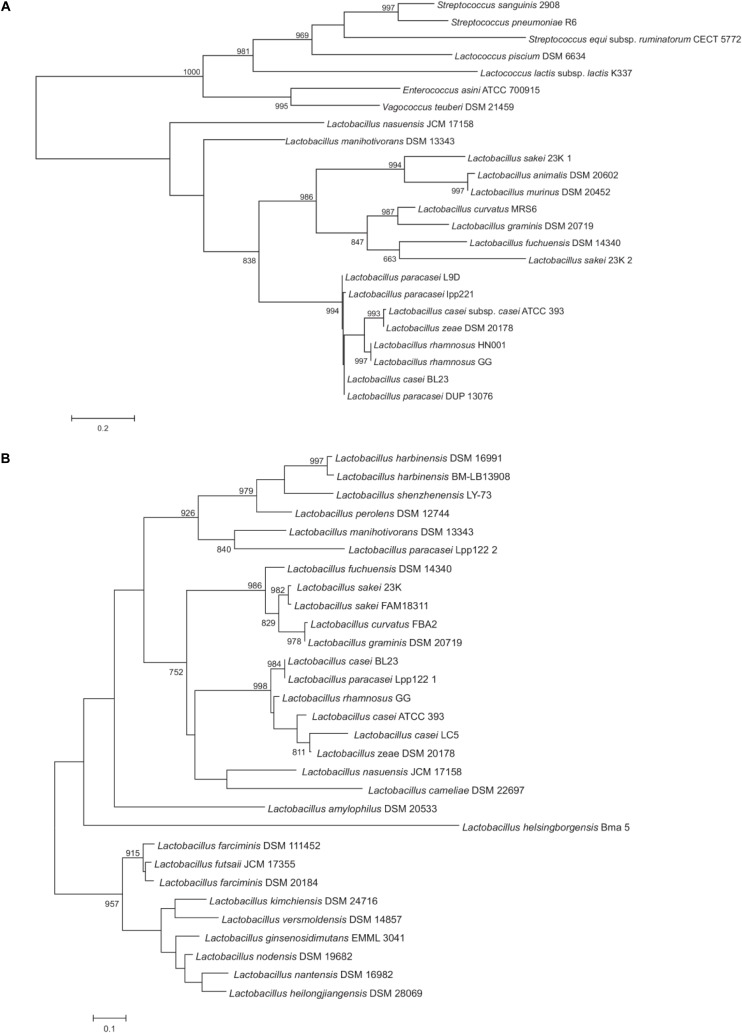
Maximum likelihood phylogenetic trees of representative P40 **(A)** and P75 **(B)** protein sequences. The trees have been arbitrarily rooted to facilitate its visualization. Support values higher than 750 for the bootstrap analysis are indicated. Protein sequence codes are supplied in Supplementary Tables S3.1,S3.2.

Proteins of the P40 cluster were identified as such when having the same domain composition and a conserved sequence homology on at least 80% of the whole protein length. In general, we found similar proteins in species closely related to the *L. casei, L. paracasei, L. zeae* and *L. rhamnosus* taxonomic group (Figure 2A). P40 homologs were also found in species of the families *Enterococcaceae* and *Streptococcaceae*. It seems interesting that the NlpC/P60 domain of P40 homologs shared similarity to the corresponding domain of the surface antigen of *E. faecium* SagA (Teng et al., 2003). In *Lactobacillus*, true P40 proteins are found in strains of the *L. casei*, *L. sakei* and *Lactobacillus salivarius* groups as defined by Salvetti et al. (2018). Within the *L. casei* group, P40 homologs are absent in *Lactobacillus brantae*, *Lactobacillus cameliae*, *Lactobacillus pantheris*, *Lactobacillus saniviri*, *Lactobacillus sharpeae*, and *Lactobacillus thailandensis*. The genes harbored by strains of *L. casei*, *L. paracasei*, *L. rhamnosus* and *L. zeae* constitute a well supported group suggesting that a P40 encoding gene was present in the common ancestor of these species. Apart from those, *Lactobacillus nasuensis* and *Lactobacillus manihotivorans*, which also belong to the *L. casei* group, encode P40 homologs but the lack of support of their phylogenetic positions make it difficult to determine their origin. The four species of the *L. sakei* group encode P40 homologs whereas only two species of the *L. salivarius* group encode P40 homologs. Remarkably, many strains of *L. sakei* encode two paralog proteins, one of them clustering with the other sequences from the *L. sakei* group, as expected, but the other one in a well-supported cluster with the sequences of *Lactobacillus murinus* and *Lactobacillus animalis*. The limited distribution of P40 encoding genes within species of the *L. salivarius* group may suggest a possible horizontal gene transfer of this gene from *L. sakei* to the last common ancestor of these two closely related species. However, the presence of two paralogs encoding P40 is quite anomalous, since no other species harbor two P40 encoding genes. Additional data would be required to settle this point. The shape of this tree does not suggest that P40 could derive from horizontal transfer from streptococci or enterococci to lactobacilli or *vice versa*. As it is, we hypothesize that a P40 encoding gene was present in the last common ancestor of *Enterococcaceae*, *Lactobacillaceae* and *Streptococcaceae*. The current distribution of these genes would be mostly explained by lineage-specific gene losses.

When applied to P75 proteins, the criterion of selection used above for P40 proteins was only fulfilled by P75 homologs of the *L. casei* and *L. sakei* groups. Therefore, to gain insight into the phylogenetic position of P75, all sequences retrieved in the PSI-BLAST search from species belonging to *Lactobacillaceae* were included in the analysis. Proteins like P75 do not have a defined consensus domain at the amino terminal region and, in fact, there is a great dispersion in sequence among them, hence, the C-terminal NlpC/P60 domain will have a fundamental weight in the phylogenetic analysis. On the basis of the homology of the NlpC/P60 domain, many other proteins could have been included in the comparison, like some enterococcal proteins, P45 and TrsG from *L. casei* and other species, but this did not add any significant information to existing trees (Anantharaman and Aravind, 2003). As previously observed for P40, P75 was found within the *L. casei* group in *L. casei*, *L. paracasei*, *L. rhamnosus* and *L. zeae*. *L. nasuensis* and *L. cameliae* also encoded putative P75 proteins (although similarity is limited to the N-terminal and C-terminal parts) whereas the corresponding gene is absent in *L. brantae*, *L. pantheris*, *L. saniviri*, *L. sharpeae*, and *L. thailandensis* (Figure 2B). P75 homologs were also present in all species of the *L. sakei* group, as previously observed for P40 (Figure 2A). Both groups form a well-supported cluster. However, phylogenomic analyses (Sun et al., 2015; Salvetti et al., 2018) do not place these two groups as sister groups and the presence of more distantly related P75 proteins in other groups of lactobacilli make it difficult to accept that evolution of P75 in lactobacilli is explained by vertical inheritance and lineage-specific gene losses. For example, the *L. perolens* group is a sister group of the *L. casei* group, however, their corresponding sequences are more distantly related than those of the *L. sakei* group (Figure 2B). Unfortunately, lack of support in deeper nodes of the tree precludes establishing reliably the relationships between these clusters. Interestingly, *L. paracasei* Lpp122 harbors a paralog encoding a putative P75 protein that clusters with the corresponding gene of *L. manihotivorans*. These two sequences form a well-supported cluster with sequences of species of the *L. perolens* group (Figure 2B). This arrangement suggests a horizontal gene transfer from a member of the *L. perolens* group. In summary, the analysis indicates that P75 proteins of *L. casei* and *L. sakei* groups share a common origin but it cannot be ascertained whether vertical inheritance or horizontal gene transfer would explain the current distribution of these genes.

### Cloning and Purification of P40, P75 and N-Terminal Domains

The complete mature proteins (P40T and P75T) were overexpressed in *E. coli* BL21 by cloning the corresponding genes (LCABL_00230, LCABL_02770) of *L. casei* BL23 in the expression vector pQE80e. PCR oligonucleotides were designed to allow in frame fusion of the gene encoding the mature protein (excluding the secretion signal) to the RGS-His epitope encoding region in pQE80e.

NlpC/P60 domains are conserved in proteins of different bacterial taxons. Hence, in order to obtain highly specific polyclonal antibodies recognizing P40 and P75, N-domains of both proteins were expressed in *E. coli* and purified. P40 N-domain (P40N) included the Cwl0(COG3883) domain (residues 74 to 211) and in the case of P75, the N-domain spanned (P75N) the complete peptide sequence upstream NlpC/P60 domain, because there was no domain description in databases associated to that region (Supplementary Figure S1A).

**FIGURE 3:**
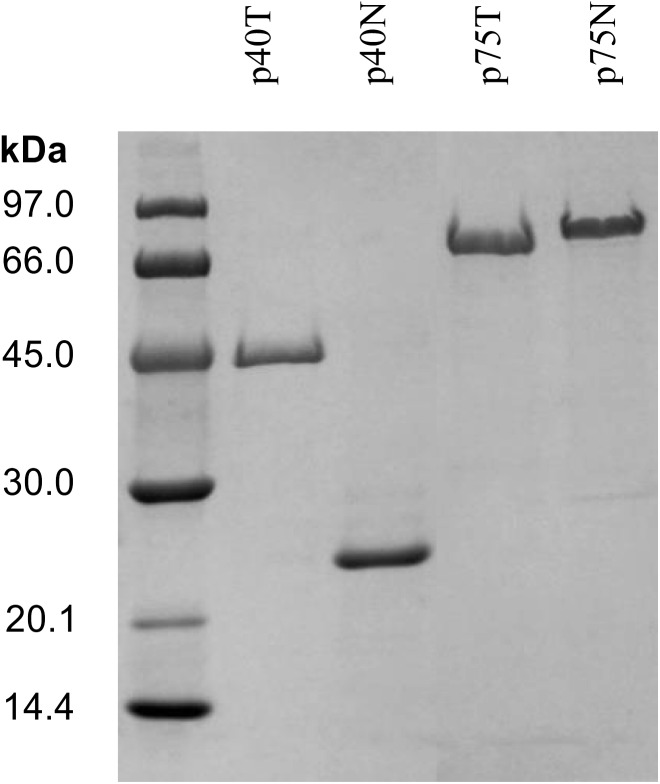
SDS-PAGE of the NiNTA purified his-tagged proteins P40 (P40T) and P75 (P75T) and their respective amino terminal domains, P40N and P75N. In order to reduce the figure’s size, part of the gel has been omitted. The complete gel is shown in Supplementary Figure S3.

The purified proteins were analyzed by SDS-PAGE and the resulting electrophoretic migration rendered apparent sizes larger than their predicted molecular mass (P40T, 42.341 kDa and P40N, 19.562 kDa), an effect that was very pronounced in the case of P75T and P75N (P75T, 49.620 kDa and P75N, 35.886 kDa), where their migration corresponded to molecular sizes over 70–80 kDa (Figure 3). This anomaly had been previously observed (Yan et al., 2007; Bäuerl et al., 2010) and could be related to its high polarity, but most probably to a very relaxed configuration of the N-terminal region, as suggested by structural predictions (Supplementary Figure S4). The homologous P75 protein of *L. rhamnosus* GG was shown to be glycosylated, which could influence its migration in PAGE, however, *L. casei* BL23 P75 -hence P75T and P75N- lack the O-glycosylation region previously described (Lebeer et al., 2012).

### Localization, Mutant Complementation and Lytic Activity of P40 and P75

**FIGURE 4:**
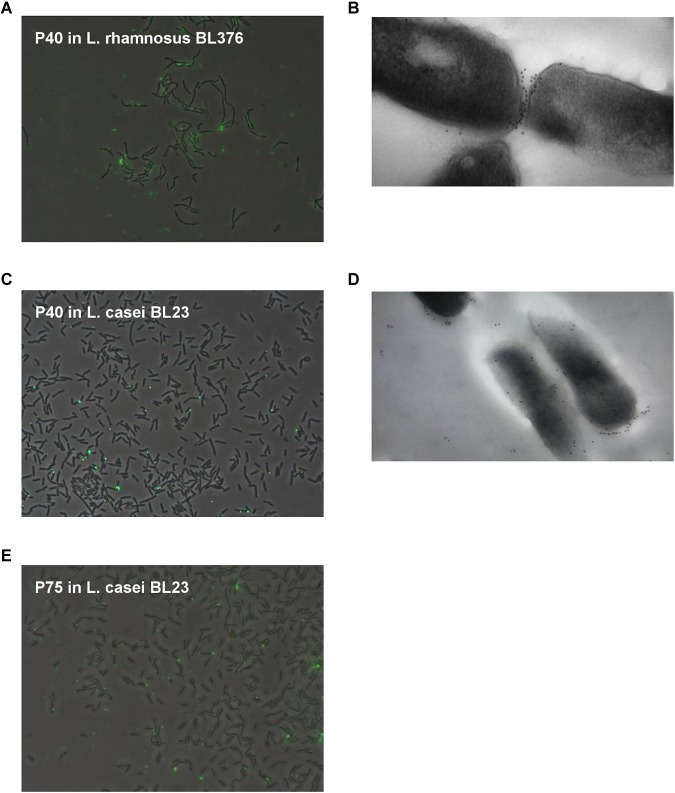
Localization of P40 in *L. casei* BL23 and *L. rhamnosus* GG with anti-P40N antibodies, and P75 in *L. casei* BL23 with anti-P75N antibodies. **(A–C)** Show random epifluorescence microscopic fields of Alexa immune labeled P40 on *L. casei* BL23 and *L. rhamnosus* BL376 (LGG); **(E)** of P75 in *L. casei* BL23; and **(B,D)** show TEM of *L. casei* BL23 and *L. rhamnosus* BL376 (LGG) subject to immunogold staining of P40.

Immunofluorescence observations showed that P40 and P75 were predominantly detected at the septum and polar regions in *L. casei* BL23 (Figure 4). When we tested P40 in the closely related bacterium *L. rhamnosus* GG, it was localized apically but a clear observation was difficult as it was possibly associated to the exopolysaccharide secreted by this strain, as can be observed both in epifluorescence and TEM (Figure 4).

**FIGURE 5:**
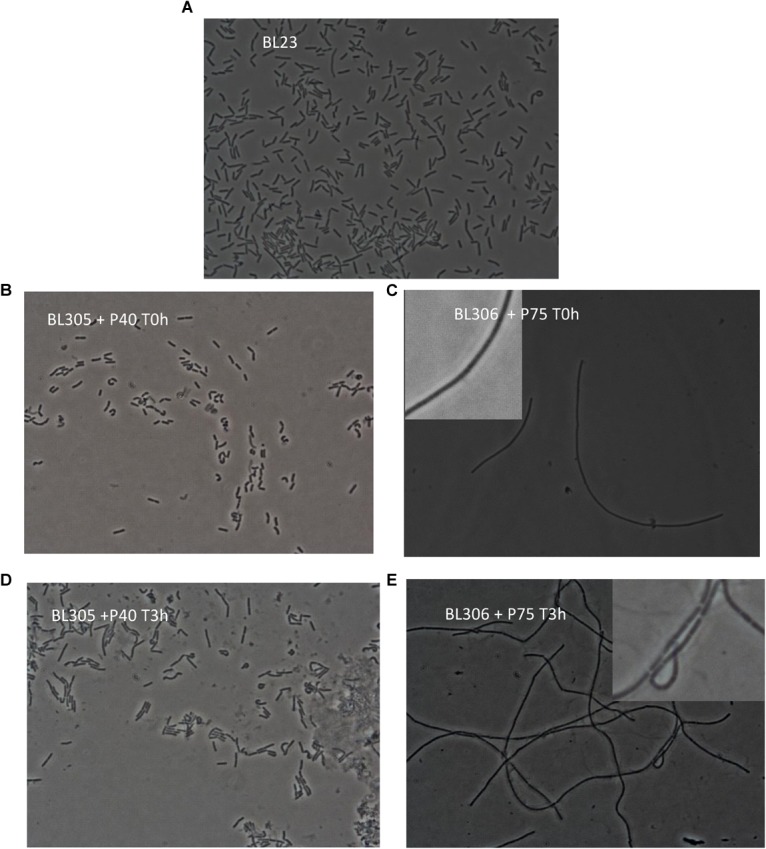
Mutant complementation with purified P40 and P75. Morphological observation by phase contrast transmission microscopy (objective × 100) of *L. casei* BL23 **(A)**, *L. casei* P40 knock down mutant, *cmu*A (BL305) incubated for 0 h **(B)** and 3 h **(D)** with purified P40 (final concentration of 50 μg/ml), and *L. casei* P75 knock down mutant, *cmu*B (BL306) incubated for 0 h **(C)** and 3 h **(E)** with purified P75 (final concentration of 50 μg/ml) and as indicated in the panels. **(C,E)** Show enlarged details for a better distinction of nascent septa.

In a previous work, P40 (BL305) and P75 (BL306) defective *L. casei* BL23 mutant strains were obtained (Bäuerl et al., 2010). Both mutants had distinctive morphologies associated to a possibly abnormal cell division process. Cell suspensions of both mutants were incubated with P40 and P75 (final concentration of 50 μg/ml) for 0 to 3 h and observed under the microscope (Figure 5). After only 3 h incubation with P40, apparent cell morphology of mutant BL305 reverted significantly to the wild type, and although BL306 typical extremely long rods persisted after 3 h incubation with purified P75, phase contrast evidenced the primordial formation of septa. These complementation assays and their localization suggest that P40 and P75 are involved in septation and separation of daughter cells in *L. casei* BL23.

Given the relative similarity of P40 and P75 to putative bacterial lysins (see below) and since these two proteins are found in the extracellular medium, their (auto)lytic activity was assayed separately and together in *L. casei* BL23 and against gram positive competitors/pathogens such as *Enterococcus faecalis*, *Listeria monocytogenes* and *Staphylococcus aureus*. Different conditions were used but no significant effect could be found in cell suspensions adjusted at different pH values even when P40 and P75 treatment was followed by a detergent (Triton x100) treatment (Supplementary Figure S5). The only noticeable effect was an increase in OD promoted by P75 in *L. casei* BL23 and by both enzymes together in *L. casei* and *E. faecalis*, suggesting that they may promote cell division, in agreement with the microscopic observations (Figure 4, 5).

### Detection of P40 and P75 in the *L. casei/paracasei/rhamnosus* Taxon and Other Lactobacilli

**FIGURE 6:**
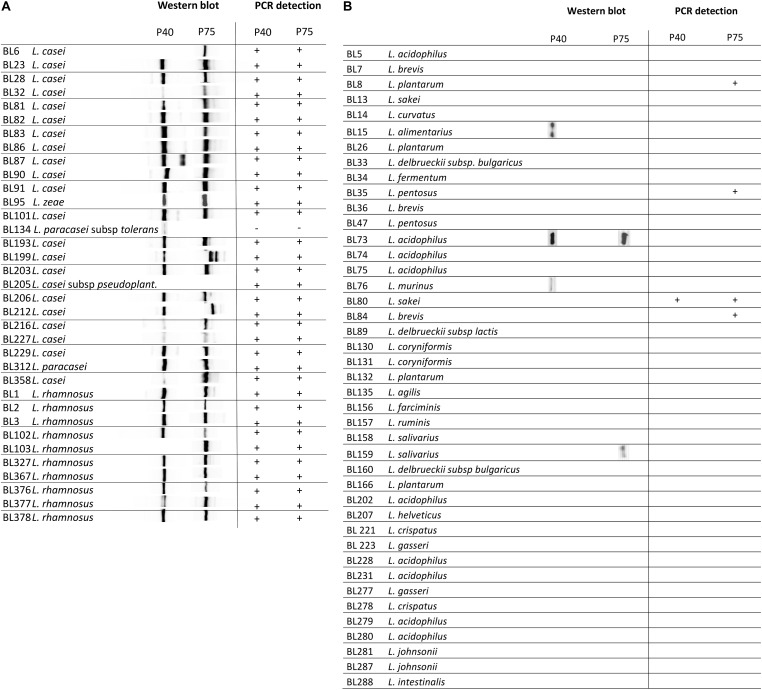
Diagram summarizing immunoblot signals (Western blot) obtained with anti-P40N and anti-P75N from overnight culture supernatants of the designated strains. **(A)** Shows a group of strains belonging to the taxon *Lactobacillus casei/paracasei/rhamnosus* and **(B)** representative strains of the genus *Lactobacillus* from the laboratory’s collection.

Homology searches indicated that most Firmicutes, also the *Lactobacillaceae* family, normally harbor more than one gene encoding proteins containing the NlpC/P60(CHAP) C-terminal domain (Layec et al., 2008). Our protein comparisons suggested that amino acid sequences of this group rendered distinctive clusters that grouped P40 and P75 separately from other CHAP-containing proteins and also found that the C-terminal NlpC/P60 domains were better conserved than the N-terminal part (see above). For this reason, N-terminal domains of both proteins were cloned as His-tag fusions and purified to obtain antibodies that were used in order to specifically recognize P40 and P75 from *L. casei/rhamnosus* strains. Initial results were very encouraging because a single band was detected with each antibody in culture supernatants of *L. casei* BL23 (Figure 6A). Then, culture supernatants of the *Lactobacillus* laboratory’s collection (Table 1) were tested by Western blot with anti-P40N and anti-P75N antibodies. Most lactobacilli belonging to *L. casei, L. zeae* and *L. rhamnosus* species showed Western blot bands that coincided with the P40 and P75 migrations in SDS-PAGE – with the exception of *L. casei* subsp *tolerans* BL134-. In a few cases, more than one band or a band of smaller molecular size were detected with anti-P40N (BL87, BL90) or anti-P75N (BL199, BL212). Strain *L. casei* subsp. *pseudoplantarum* BL205 did not show any band and P40 was missing under the conditions assayed in *L. casei* BL6 and *L. rhamnosus* BL103. Inspection of the genome of *L. casei* BL6 (ATCC393) reveals however, that this strain harbors a gene encoding a “CHAP domain-containing protein” (WP_025013825.1) with 85% identity to BL23 P40 (WP_003572828.1). In some cases, P40 may not have been detected in the Western blots because of its instability in the culture supernatant after 24 h incubation, as will be pointed out below. Very few *Lactobacillus* strains belonging to other species showed bands corresponding to P40 and P75 with these antibodies (Figure 6B), like *L. acidophilus* BL73, *L. alimentarius* BL15, *L. murinus* BL76 and *L. casei* subsp. *tolerans* (only P40) and *L. salivarius* BL159 (only P75).

When polyclonal antibodies raised against the purified complete proteins P40 and P75 (Yan et al., 2007) were used for Western blot of non *L. casei/rhamnosus* lactobacilli different bands were visualized, remarkably in all strains of *L. plantarum*, some of *L. acidophilus, L. pentosus, L. sakei, L. brevis, L. coryniformis* and *L. agilis* (Supplementary Figure S6). This confirmed that the antibodies obtained here against the N-terminal domain of P40 and P75 gained specificity.

### PCR Detection of P40 and P75 Encoding Genes in Lactobacilli

The similarity at DNA level of the corresponding genes in the species of *L. casei, L. paracasei, L. zeae* and *L. rhamnosus* allowed the design of oligonucleotides targeted to different conserved sequences (Supplementary Tables S4, S5 and Figure 6A,B) that were used to test the specific amplification of *cmu*A and *cmu*B by PCR. The oligonucleotide combinations that rendered reproducible positive bands in all *L. casei/paracasei/rhamnosus* strains of the laboratory’s collection are described in Table 2. The only exception was strain *L. casei* subsp *tolerans* BL134 as occurred in Western blots. P40-encoding gene could be unequivocally amplified with primers P40carh-f1/P40carh-r yielding amplicons of about 278 nt (Table 2). The P75 encoding gene was identified by PCR with P75carh-f and P75carh-r obtaining a band of 138 nt. In other *Lactobacillus* species both genes were only amplified in *L. sakei* BL80. The rest of *Lactobacillus* strains tested yielded no amplification bands, excepting the P75 specific PCR in *L. plantarum* BL8, *L. pentosus* BL35 and *Lactobacillus delbrueckii* subsp *lactis* BL89.

### Detection of P40 and P75 in Fermented Dairy Products

**FIGURE 7:**
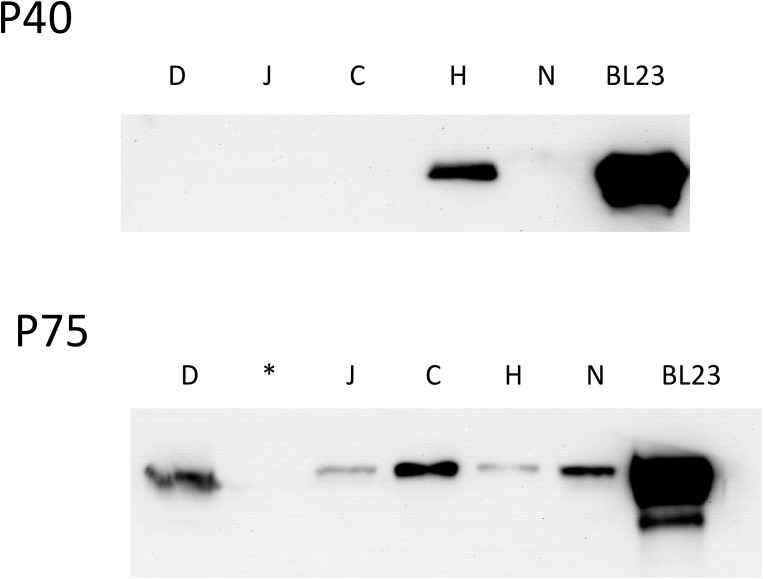
Western blot of the supernatant of different commercial dairy products containing *Lactobacillus casei* (D, J, C, H, N). Product names have been replaced by letter codes to avoid conflict of interests. In position 6 BL23 supernatant was included as a reference. (^∗^) In the image of P75 blot, the second line was not loaded with sample.

Different commercial products found in local supermarkets that contained in their ingredients strains of *L. casei* were used to detect P40 and P75. In order to avoid possible interference of gelling agents, five drinkable products were selected (D, C, J, H, N) and their clear fraction and pellets were subjected to Western blot (Figure 7). P40 could only be detected in the supernatant of one of the products (H) while P75 was found in four of them (D, J, H, N). Four other fermented dairy products that did not contain *L. casei* were also tested but did not show any band on both assays (data not shown). Binding to the food matrix components or instability of P40 during the long shelf life of those products (at least 28 days) could explain why it was hardly detected.

## Discussion

Proteins P40 and P75 were first described in the biologically active fraction of *L. rhamnosus* GG conditioned medium that prevented cytokine-induced apoptosis in human and mouse intestinal epithelial cells (Yan et al., 2007). Purified P40 and P75 from *L. rhamnosus* GG and *L. casei* BL23 were shown to stimulate *in vitro* phosphorylation of EGFR and Akt, hence blocking apoptosis (Yan et al., 2007; Bäuerl et al., 2010). Yet there are evidences suggesting other biological activities of this family of proteins on the basis of their PGN hydrolase activity, as bacterial PGN fractions have specific surface receptors in eukaryotic cells and distinctive innate immune system stimulatory effects (Humann and Lenz, 2009). In fact, a P40 related protein, *Enterococcus faecium* SagA, promoted *Salmonella typhimurium* tolerance in *Caenorhabditis elegans* through its hydrolase activity, as it generated PGN fragments that activated the host immune pathways through TLR-1 (Rangan et al., 2016). As P40 and P75 are PGN hydrolases, their enzymatic activities may constitute an additional factor contributing to their functional properties *in vivo*. Both proteins have a C-terminal NlpC/P60 domain, a widely distributed domain in bacteria and bacteriophages, which is also present even in eukaryotic regulators, proteins of poxviruses and animal RNA viruses [for reviews, see (Anantharaman and Aravind, 2003; Bateman and Rawlings, 2003)]. P40 and P75 belong to an important group of cell wall degrading enzymes with different N-terminal regions. Layec et al. (2008) described in Firmicutes 12 different subgroups. P40 proteins found in *L. casei, L. paracasei, L. zeae* and *L. rhamnosus* constitute a well-supported cluster. Taxonomically close species, like those included in the *L. sakei* and *L. salivarius* clusters (Salvetti et al., 2018) also encode P40 homologs. The phylogenetic analysis suggests that an ancestral P40 encoding gene was present in the last common ancestor of *Enterococcaceae*, *Lactobacillaceae* and *Streptococcaceae*, with lineage-specific gene loss explaining its current distribution. The case of P75 was very different, as there is not a clearly defined N-terminal domain in P75 homologs, so the NlpC/P60 domain had a greater weight in the analysis. P75 related proteins were only present in the *L. casei* and *L. sakei* groups, but there was lack of support for deep nodes in those taxonomically close bacterial groups and in addition, *L. perolens* P75 was a sister group of the *L. casei* group. This analysis indicated that P75 proteins of *L. casei* and *L. sakei* groups share a common origin, but the results obtained did not allow obtaining a clear picture of their evolutionary history.

When analyzing the nucleotide sequence of *cmu*B two highly conserved tandem repeats of 18 nucleotides were clearly identified with four strain-specific copy number variations (alleles: *cmu*B1, *cmu*B2, *cmu*B3, *cmu*B4). A similar case of TR in a PGN hydrolase was described in the *cse* gene of *S. thermophilus* (Borges et al., 2006), although this was a more complex scenario with a mosaic of 11 different TR sequences grouped in larger repeated sequence blocks, with additional degrees of sequence degeneration. As in the case of *S. thermophilus cse*, all alleles are functional suggesting that TR protein sequences in CmuB are not essential for the activity of the enzyme, acting as spacers between the C-terminal NlpC/P60 domain and other so far uncharacterized domain(s) at the N-terminal region. TR sequences are prone to insertions and deletions (indels) providing flexibility for functional adaptation. They are frequent in surface exposed proteins that interact directly with host structures (Moxon et al., 2006), like epitopes or adhesion/invasion elements of epithelial pathogens (Zhou et al., 2014). This genetic variability translated to protein sequences only affected strains within the *L. casei/paracasei* species groups and had no relevance in the phylogenetic analysis performed. The presence of the microsatellites in *cmu*B was only detected in strains considered as *L. paracasei*, this discovery constitutes the basis of an interesting taxonomical feature to be further explored.

In previous studies, P40 and P75 were recognized by anti-P40 antibodies in a large number of *L. casei/paracasei/rhamnosus* strains (Yan et al., 2007; Bäuerl et al., 2010) suggesting those antibodies had a broad specificity possibly recognizing P40 and P75 similar surface epitopes. It is shown here that antibody specificity was much improved by immunizing rabbits with the purified N-terminal regions of both proteins. The specific detection of P40 and P75 in *L. casei/paracasei/rhamnosus* strains was successfully achieved using polyclonal anti-P40N and anti-P75N rabbit antibodies obtained, and not in other *Lactobacillus* species, with only a few exceptions. Absence of Western blot signal in some *L. casei/paracasei/rhamnosus* strains may be due to the culture conditions used, or to the fact that homologous genes are not found in some strains, possibly because the activity of those proteins may be efficiently replaced and complemented by other NlpC/P60 proteins. Frequently, Firmicutes genomes contain more than two genes encoding NlpC/P60 PGN hydrolases. In addition to P40 and P75 (WP_003572828.1, WP_012490875.1), *L. casei* BL23 genome contains genes encoding at least two additional NlpC/P60 C-terminal domain proteins (WP_012491782.1, WP_012491062.1) not mentioned in our study. *Lactobacillus plantarum* WCFS1, another well known probiotic strain, encodes five NlpC/P60 C-terminal putative proteins that do not belong to the P40 or P75 families (Supplementary Table S1), four of them (LytA, LytB, LytC, LytD) recently studied in detail (Duchêne et al., 2019).

In the case of P75, subcloning of the N-terminal domain showed that this region is responsible for the unusual migration in SDS-PAGE of the whole protein. P40 and P75 have always been detected in the culture supernatants. This work showed that P40 and P75 are localized or at least bind to the polar regions in BL23, and that purified proteins would complement specific mutants where *cmu*A and *cmu*B had been inactivated. Microscopic images (Figure 4, 5) suggest that P40 may participate in early cell division stages and perimetric cell growth and P75 may be required in septum formation and cell separation. Lytic activity has been described in other NlpC/P60 proteins in lactobacilli (Liu et al., 2015), but P40 and P75 did not show such activity in our experiments suggesting that cell wall remodeling does not necessarily imply a lytic or autolytic activity (Vermassen et al., 2019).

## Conclusion

This work has defined genetic characteristics of the genes encoding P40 and P75 in *L. casei/paracasei* strains. These antiapoptotic proteins are actually required by the bacterial cells for growth and cell division and the phylogenetic analysis suggested that they are found in *L. casei/paracasei/rhamnosus* (*L. casei* phylogenomic group) and close species like *L. sakei*. P40 likely evolved by vertical inheritance, while in some cases horizontal transfer may have also participated in the inheritance of P75 proteins. In addition to these findings, we have developed tools that specifically recognize *L. casei* phylogenomic group, such as the anti-P40N and anti-P75N antibodies detecting P40 and P75 in *L. casei/paracasei/rhamnosus* strains, also suitable for commercial fermented dairy products, and PCR primers that specifically recognize *L. casei/paracasei/rhamnosus* strains.

## Data Availability

All datasets for this study are included in the manuscript and the Supplementary Files.

## Ethics Statement

This study was carried out in accordance with the recommendations of the European Union Law and 2010/63/EU and the Spanish Government RD 53/2013 on the protection of animals used for scientific purposes, name of committee. The protocol was approved by the Ethical Committee of University of Valencia and had the corresponding authorization of the Government of the Comunitat Valenciana (2014/VSC/PEA/00197).

## Author Contributions

All authors have contributed significantly to the development of this work. CB cloned, purified, and produced the antibodies and initiated the western blot series. GA performed the western blots, microscopic preparations, and DNA sequence alignments. SS-C, AM-B, and NN-L initiated all the cloning series until the proteins could be expressed in *E. coli* and initiated the phylogenetic analysis and western blots. JC-M contribution was crucial to achieve cloning and microscope preparations. MZ-C reorganized and performed the final phylogenetic analysis and evolutionary conclusions. SS contributed to the supervision and manuscript preparation. GP-M supervised and financed all the experiments and carried out the genetic analysis, writing up and figure preparation for the submission.

## Conflict of Interest Statement

The authors declare that the research was conducted in the absence of any commercial or financial relationships that could be construed as a potential conflict of interest.

## Footnotes

1 https://blast.ncbi.nlm.nih.gov/Blast.cgi

2 http://tcoffee.crg.cat

3 http://molevol.cmima.csic.es/castresana/Gblocks_server.html

4 http://www.atgc-montpellier.fr/phyml
