# Supplementary material for: P40 and P75 Are Singular Functional Muramidases Present in the Lactobacillus casei /paracasei/rhamnosus Taxon
Source: Front Microbiol. 2019 Jun 26;10:1420. doi: 10.3389/fmicb.2019.01420 (PMC6607858; doi:10.3389/fmicb.2019.01420)
Supplement: Supplementary file 1 [file Presentation_1.PPTX]

## Slide 1
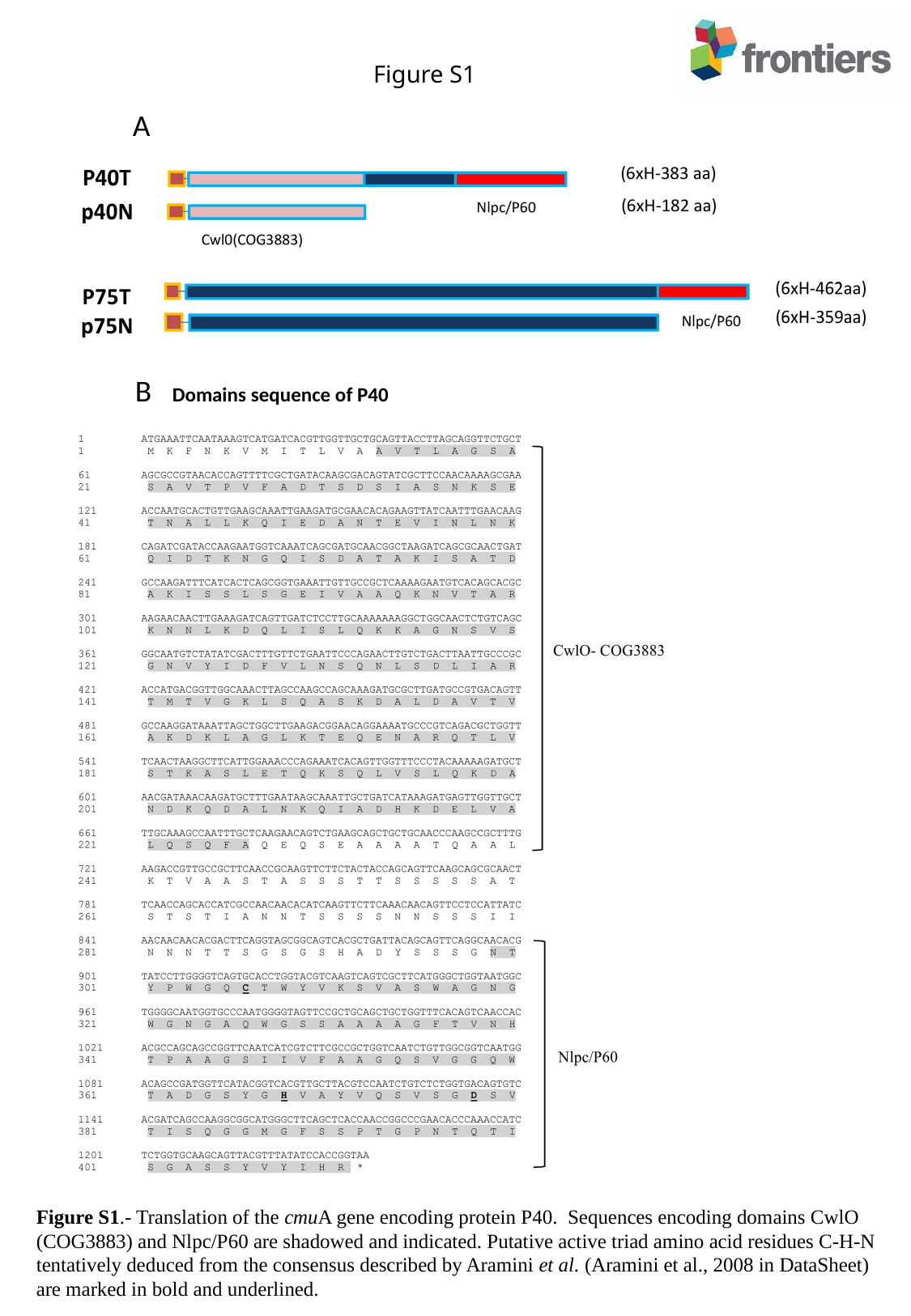

# Figure S1
A
B Domains sequence of P40
Figure S1.- Translation of the cmuA gene encoding protein P40. Sequences encoding domains CwlO (COG3883) and Nlpc/P60 are shadowed and indicated. Putative active triad amino acid residues C-H-N tentatively deduced from the consensus described by Aramini et al. (Aramini et al., 2008 in DataSheet) are marked in bold and underlined.

## Slide 2
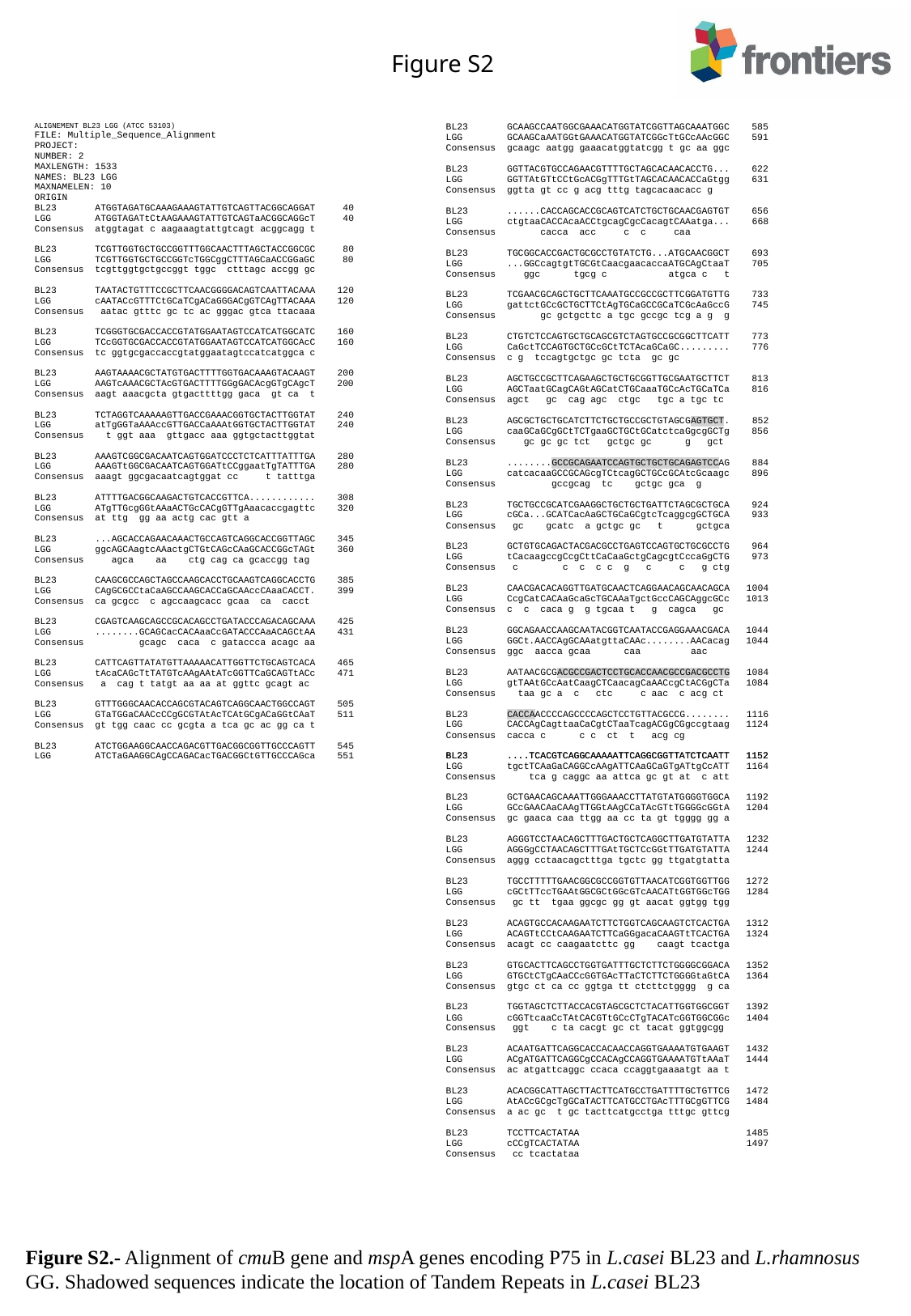

# Figure S2
Figure S2.- Alignment of cmuB gene and mspA genes encoding P75 in L.casei BL23 and L.rhamnosus GG. Shadowed sequences indicate the location of Tandem Repeats in L.casei BL23

## Slide 3
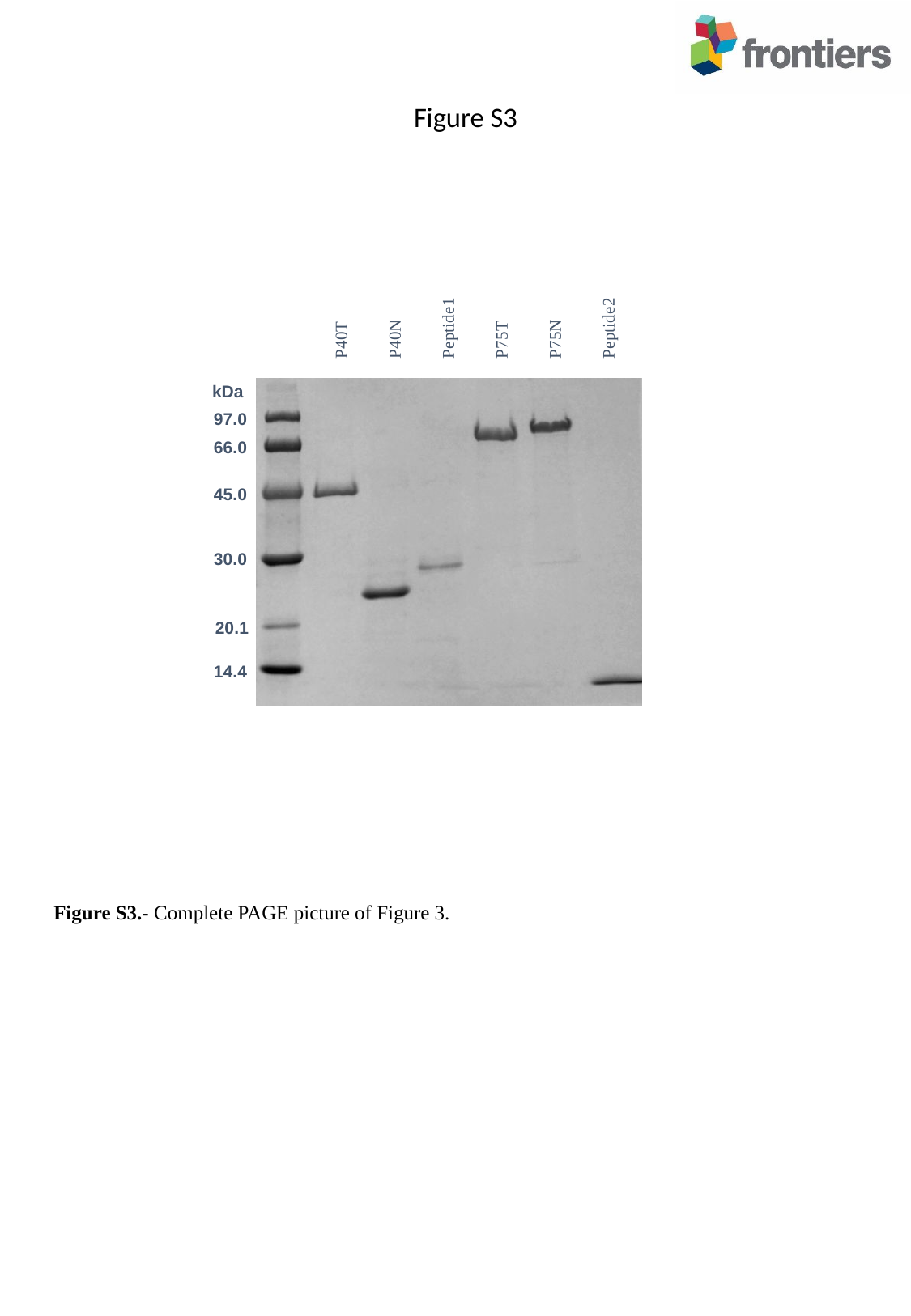

Figure S3
P40T
P40N
Peptide1
P75T
P75N
Peptide2
kDa
97.0
66.0
45.0
30.0
20.1
14.4
Figure S3.- Complete PAGE picture of Figure 3.

## Slide 4
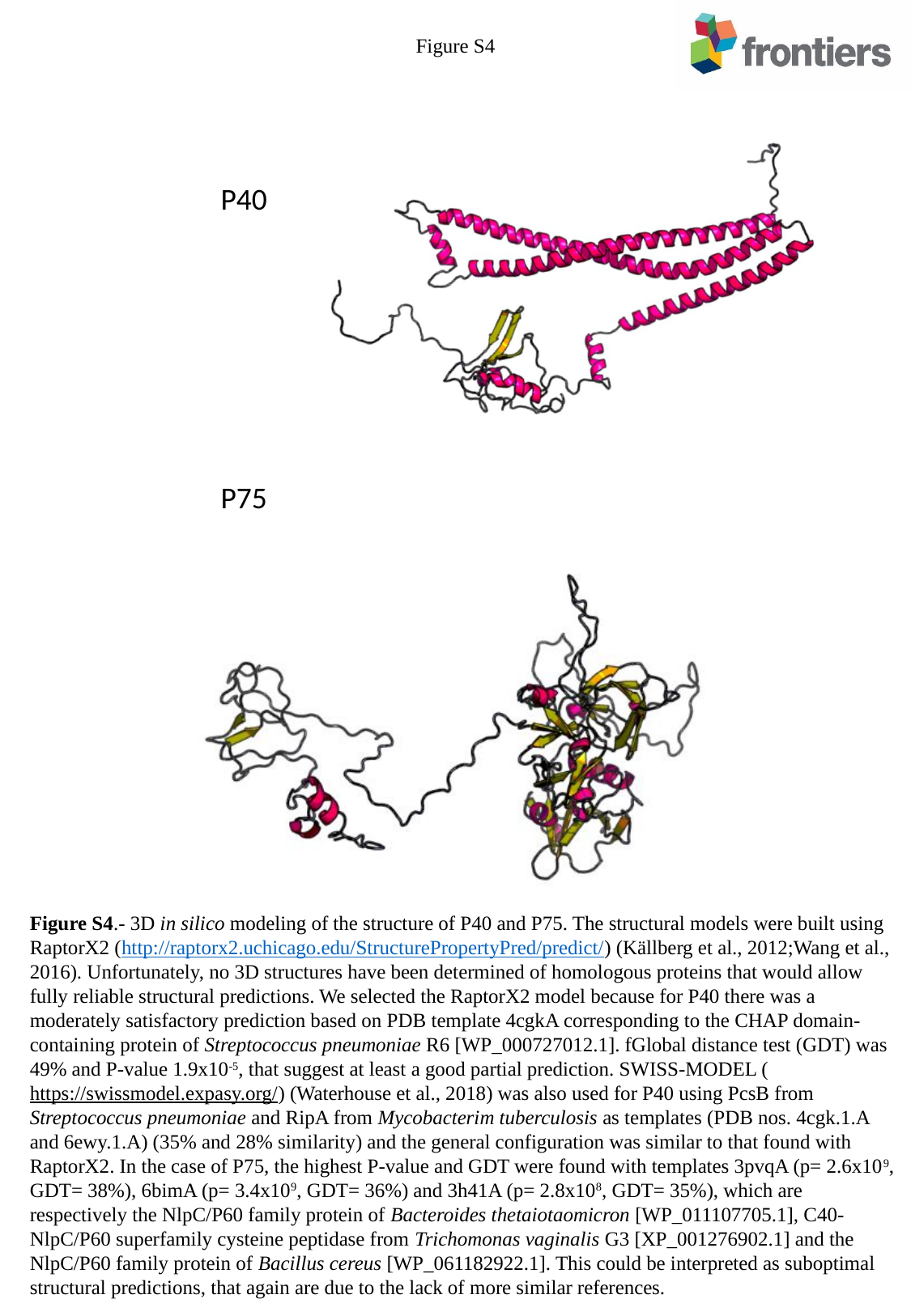

# Figure S4
P40
P75
Figure S4.- 3D in silico modeling of the structure of P40 and P75. The structural models were built using RaptorX2 (http://raptorx2.uchicago.edu/StructurePropertyPred/predict/) (Källberg et al., 2012;Wang et al., 2016). Unfortunately, no 3D structures have been determined of homologous proteins that would allow fully reliable structural predictions. We selected the RaptorX2 model because for P40 there was a moderately satisfactory prediction based on PDB template 4cgkA corresponding to the CHAP domain-containing protein of Streptococcus pneumoniae R6 [WP_000727012.1]. fGlobal distance test (GDT) was 49% and P-value 1.9x10^-5^, that suggest at least a good partial prediction. SWISS-MODEL (https://swissmodel.expasy.org/) (Waterhouse et al., 2018) was also used for P40 using PcsB from Streptococcus pneumoniae and RipA from Mycobacterim tuberculosis as templates (PDB nos. 4cgk.1.A and 6ewy.1.A) (35% and 28% similarity) and the general configuration was similar to that found with RaptorX2. In the case of P75, the highest P-value and GDT were found with templates 3pvqA (p= 2.6x10^9^, GDT= 38%), 6bimA (p= 3.4x10^9^, GDT= 36%) and 3h41A (p= 2.8x10^8^, GDT= 35%), which are respectively the NlpC/P60 family protein of Bacteroides thetaiotaomicron [WP_011107705.1], C40-NlpC/P60 superfamily cysteine peptidase from Trichomonas vaginalis G3 [XP_001276902.1] and the NlpC/P60 family protein of Bacillus cereus [WP_061182922.1]. This could be interpreted as suboptimal structural predictions, that again are due to the lack of more similar references.

## Slide 5
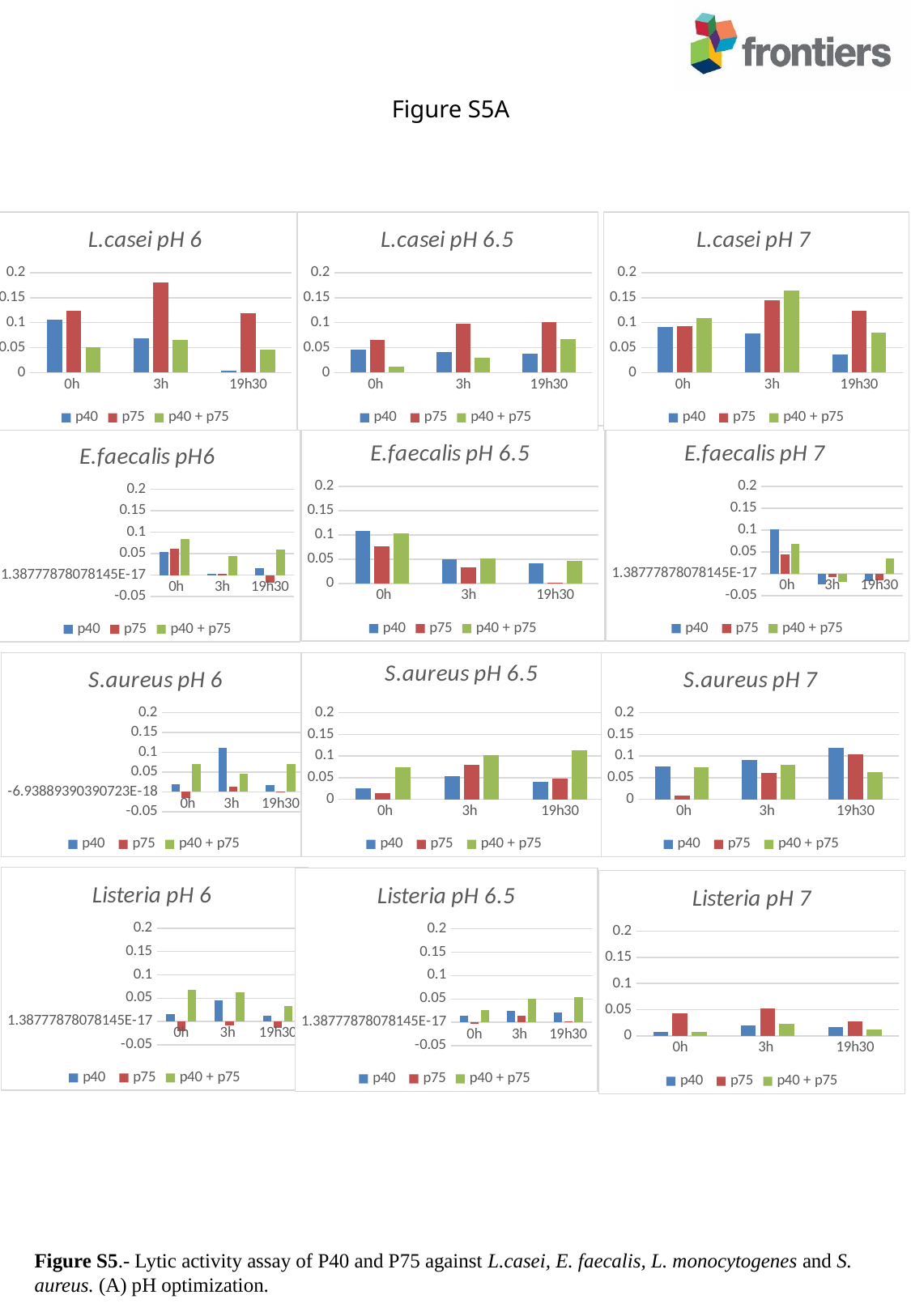

# Figure S5A
### Chart: L.casei pH 6
| Category | | | |
|---|---|---|---|
| 0h | 0.10560000000000003 | 0.12405000000000002 | 0.0514 |
| 3h | 0.06905 | 0.18014999999999998 | 0.0648 |
| 19h30 | 0.0038499999999999923 | 0.11945 | 0.045499999999999985 |
### Chart: L.casei pH 6.5
| Category | | | |
|---|---|---|---|
| 0h | 0.045650000000000024 | 0.06520000000000001 | 0.012650000000000022 |
| 3h | 0.04050000000000001 | 0.09820000000000001 | 0.02975 |
| 19h30 | 0.03759999999999997 | 0.10164999999999999 | 0.06664999999999999 |
### Chart: L.casei pH 7
| Category | | | |
|---|---|---|---|
| 0h | 0.0912 | 0.0937 | 0.1086 |
| 3h | 0.07879999999999998 | 0.14530000000000004 | 0.16355000000000003 |
| 19h30 | 0.03544999999999998 | 0.12409999999999999 | 0.08015 |
### Chart: E.faecalis pH 6.5
| Category | | | |
|---|---|---|---|
| 0h | 0.10805000000000001 | 0.07700000000000001 | 0.10269999999999999 |
| 3h | 0.04995000000000002 | 0.03250000000000003 | 0.052199999999999996 |
| 19h30 | 0.04200000000000001 | 0.0007999999999999952 | 0.04619999999999999 |
### Chart: E.faecalis pH 7
| Category | | | |
|---|---|---|---|
| 0h | 0.1028 | 0.04365000000000002 | 0.06915000000000002 |
| 3h | -0.023649999999999977 | -0.0063499999999999945 | -0.01815 |
| 19h30 | -0.017300000000000038 | -0.014000000000000012 | 0.03514999999999999 |
### Chart: E.faecalis pH6
| Category | | | |
|---|---|---|---|
| 0h | 0.053300000000000014 | 0.06139999999999998 | 0.08335000000000001 |
| 3h | 0.0024500000000000077 | 0.0030999999999999917 | 0.044300000000000006 |
| 19h30 | 0.015399999999999997 | -0.01755000000000001 | 0.06039999999999998 |
### Chart: S.aureus pH 6
| Category | | | |
|---|---|---|---|
| 0h | 0.018250000000000016 | -0.014649999999999969 | 0.07050000000000003 |
| 3h | 0.11204999999999998 | 0.012700000000000017 | 0.04484999999999997 |
| 19h30 | 0.01745000000000002 | 0.0012500000000000011 | 0.0698 |
### Chart: S.aureus pH 6.5
| Category | | | |
|---|---|---|---|
| 0h | 0.026300000000000018 | 0.013650000000000023 | 0.0736 |
| 3h | 0.05319999999999997 | 0.07885 | 0.10289999999999999 |
| 19h30 | 0.040949999999999986 | 0.04694999999999999 | 0.11279999999999998 |
### Chart: S.aureus pH 7
| Category | | | |
|---|---|---|---|
| 0h | 0.07535 | 0.009449999999999986 | 0.0741 |
| 3h | 0.09005000000000002 | 0.061000000000000026 | 0.08010000000000003 |
| 19h30 | 0.11839999999999998 | 0.10379999999999998 | 0.06319999999999998 |
### Chart: Listeria pH 6
| Category | | | |
|---|---|---|---|
| 0h | 0.015300000000000008 | -0.01999999999999999 | 0.06815000000000002 |
| 3h | 0.0456 | -0.00814999999999999 | 0.06190000000000001 |
| 19h30 | 0.012749999999999984 | -0.013450000000000017 | 0.03284999999999999 |
### Chart: Listeria pH 6.5
| Category | | | |
|---|---|---|---|
| 0h | 0.014500000000000013 | -0.003149999999999986 | 0.02560000000000001 |
| 3h | 0.024049999999999988 | 0.01394999999999999 | 0.05034999999999998 |
| 19h30 | 0.021150000000000016 | 0.0027500000000000163 | 0.05335000000000002 |
### Chart: Listeria pH 7
| Category | | | |
|---|---|---|---|
| 0h | 0.0071500000000000175 | 0.04290000000000002 | 0.007349999999999995 |
| 3h | 0.020350000000000007 | 0.05224999999999999 | 0.022649999999999976 |
| 19h30 | 0.016100000000000003 | 0.028299999999999992 | 0.012249999999999983 |Figure S5.- Lytic activity assay of P40 and P75 against L.casei, E. faecalis, L. monocytogenes and S. aureus. (A) pH optimization.

## Slide 6
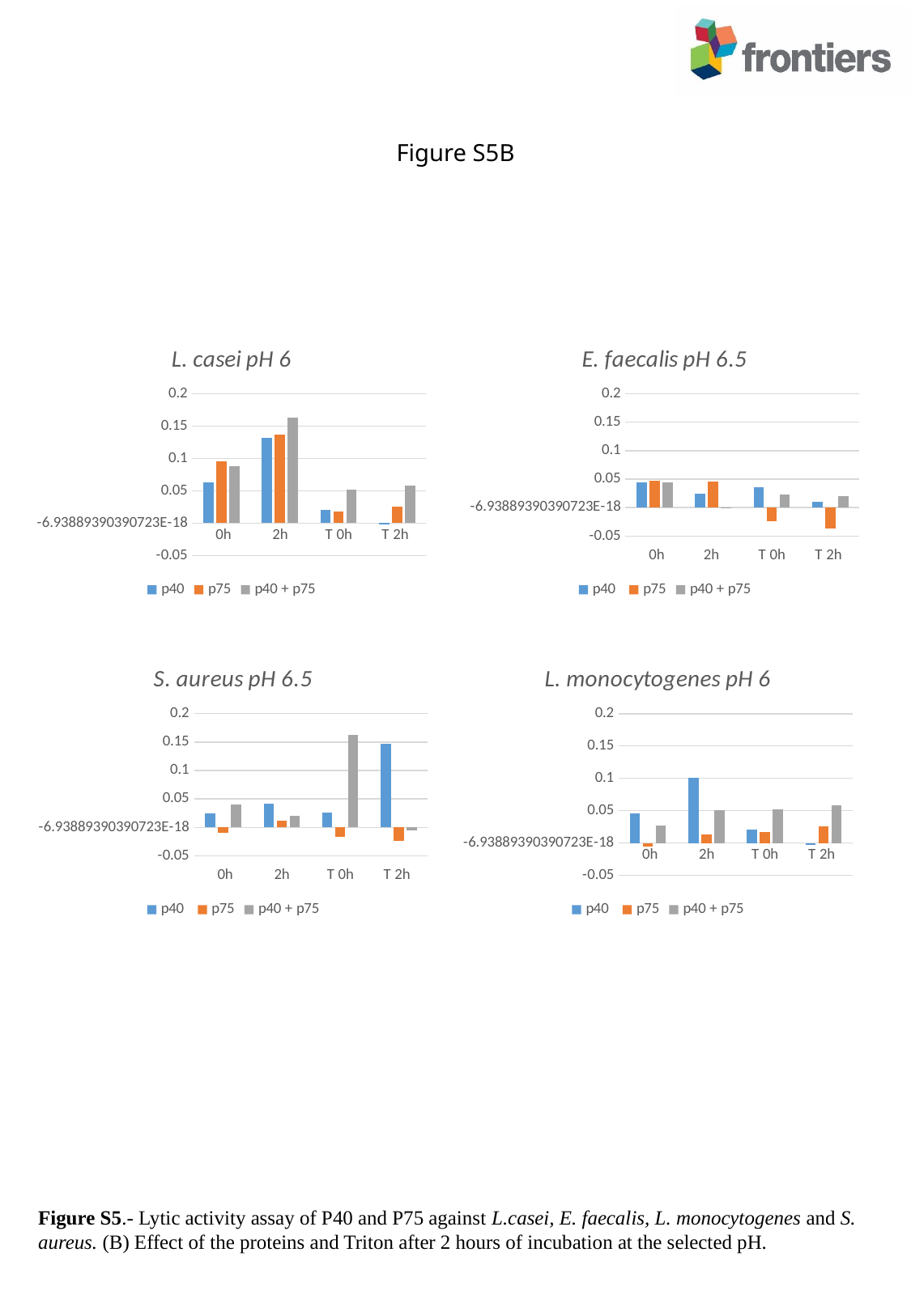

# Figure S5B
### Chart: L. casei pH 6
| Category | | | |
|---|---|---|---|
| 0h | 0.06280000000000002 | 0.09589999999999999 | 0.08865 |
| 2h | 0.13215 | 0.13725 | 0.16355 |
| T 0h | 0.021049999999999985 | 0.017549999999999982 | 0.051699999999999996 |
| T 2h | -0.0025499999999999967 | 0.025850000000000012 | 0.05770000000000003 |
### Chart: E. faecalis pH 6.5
| Category | | | |
|---|---|---|---|
| 0h | 0.0441 | 0.0464 | 0.04469999999999999 |
| 2h | 0.024249999999999994 | 0.04605000000000001 | -0.00015000000000001124 |
| T 0h | 0.03635000000000002 | -0.024399999999999977 | 0.02300000000000002 |
| T 2h | 0.010550000000000004 | -0.036599999999999994 | 0.0204 |
### Chart: S. aureus pH 6.5
| Category | | | |
|---|---|---|---|
| 0h | 0.024999999999999967 | -0.009350000000000025 | 0.03959999999999997 |
| 2h | 0.04119999999999999 | 0.011449999999999988 | 0.020400000000000015 |
| T 0h | 0.026100000000000012 | -0.016850000000000004 | 0.1621 |
| T 2h | 0.14629999999999996 | -0.02415000000000002 | -0.005750000000000005 |
### Chart: L. monocytogenes pH 6
| Category | | | |
|---|---|---|---|
| 0h | 0.045649999999999996 | -0.004999999999999977 | 0.026500000000000024 |
| 2h | 0.10095000000000001 | 0.013600000000000029 | 0.050600000000000034 |
| T 0h | 0.021049999999999985 | 0.017549999999999982 | 0.051699999999999996 |
| T 2h | -0.0025499999999999967 | 0.025850000000000012 | 0.05770000000000003 |Figure S5.- Lytic activity assay of P40 and P75 against L.casei, E. faecalis, L. monocytogenes and S. aureus. (B) Effect of the proteins and Triton after 2 hours of incubation at the selected pH.

## Slide 7
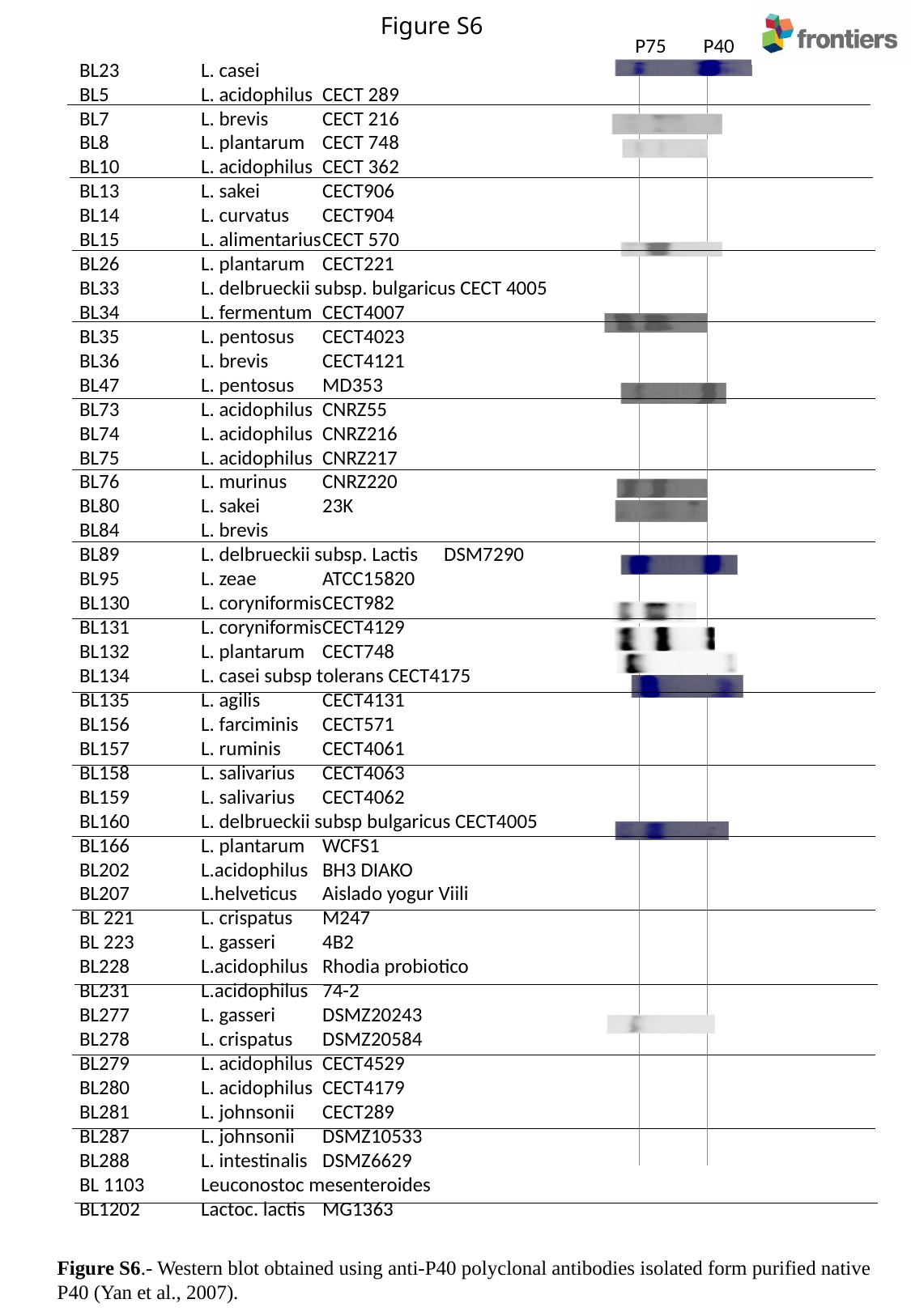

# Figure S6
	 		 P75 P40
BL23 	L. casei
BL5	L. acidophilus	CECT 289
BL7	L. brevis	CECT 216
BL8	L. plantarum	CECT 748
BL10	L. acidophilus	CECT 362
BL13	L. sakei	CECT906
BL14	L. curvatus	CECT904
BL15	L. alimentarius	CECT 570
BL26	L. plantarum	CECT221
BL33	L. delbrueckii subsp. bulgaricus CECT 4005
BL34	L. fermentum	CECT4007
BL35	L. pentosus	CECT4023
BL36	L. brevis	CECT4121
BL47	L. pentosus	MD353
BL73	L. acidophilus	CNRZ55
BL74	L. acidophilus	CNRZ216
BL75	L. acidophilus	CNRZ217
BL76	L. murinus	CNRZ220
BL80	L. sakei	23K
BL84	L. brevis
BL89	L. delbrueckii subsp. Lactis	DSM7290
BL95	L. zeae	ATCC15820
BL130	L. coryniformis	CECT982
BL131	L. coryniformis	CECT4129
BL132	L. plantarum	CECT748
BL134	L. casei subsp tolerans CECT4175
BL135	L. agilis	CECT4131
BL156	L. farciminis	CECT571
BL157	L. ruminis	CECT4061
BL158	L. salivarius	CECT4063
BL159	L. salivarius	CECT4062
BL160	L. delbrueckii subsp bulgaricus CECT4005
BL166	L. plantarum 	WCFS1
BL202	L.acidophilus	BH3 DIAKO
BL207	L.helveticus	Aislado yogur Viili
BL 221	L. crispatus	M247
BL 223	L. gasseri	4B2
BL228	L.acidophilus	Rhodia probiotico
BL231	L.acidophilus	74-2
BL277	L. gasseri	DSMZ20243
BL278	L. crispatus	DSMZ20584
BL279	L. acidophilus	CECT4529
BL280	L. acidophilus	CECT4179
BL281	L. johnsonii	CECT289
BL287	L. johnsonii	DSMZ10533
BL288	L. intestinalis	DSMZ6629
BL 1103	Leuconostoc mesenteroides
BL1202	Lactoc. lactis	MG1363
Figure S6.- Western blot obtained using anti-P40 polyclonal antibodies isolated form purified native P40 (Yan et al., 2007).
