# Supplementary material for: P40 and P75 Are Singular Functional Muramidases Present in the Lactobacillus casei /paracasei/rhamnosus Taxon
Source: Front Microbiol. 2019 Jun 26;10:1420. doi: 10.3389/fmicb.2019.01420 (PMC6607858; doi:10.3389/fmicb.2019.01420)
Supplement: Supplementary file 2 [file Data_Sheet_1.pdf]

Supplementary Material

P40 and P75 are singular functional muramidases typically found in the *Lactobacillus casei/paracasei/rhamnosus* taxon

Christine Bäuerl[1#], Gulyaim Abitayeva[2, 4#], Sebastián Sosa Carrillo[3], Ana Mencher Beltrán[1], Noemí Navarro Lleo[1], José M. Coll Marqués[1], Manuel Zúñiga Cabrera[1], Serik Shaikhin[2] and Gaspar Pérez Martinez[1*]

[#] Both authors contributed equally to the work

***Corresponding autor**:

Gaspar Pérez Martínez: gaspar.perez@iata.csic.es

1. Supplementary Figure Legends

Figure S1.- Translation of the *cmu*A gene encoding protein P40. Sequences encoding domains CwlO (COG3883) and Nlpc/P60 are shadowed and indicated. Putative active triad amino acid residues C-H-N tentatively deduced from the consensus described by Aramini *et al.* (Aramini et al., 2008) are marked in bold and underlined.

Figure S2.- Alignment of *cmu*B gene and *msp*A genes encoding P75 in *L.casei* BL23 and *L.rhamnosus* GG. Shadowed sequences indicate the location of Tandem Repeats in *L.casei* BL23.

Figure S3.- Complete PAGE picture of Figure 3.

Figure S4.- 3D *in silico* modeling of the structure of P40 and P75. The structural models were built using RaptorX2 (http://raptorx2.uchicago.edu/StructurePropertyPred/predict/) (Källberg et al., 2012;Wang et al., 2016). Unfortunately, no 3D structures have been determined of homologous proteins that would allow fully reliable structural predictions. We selected the RaptorX2 model because for P40 there was a moderately satisfactory prediction based on PDB template 4cgkA corresponding to the CHAP domain-containing protein of *Streptococcus pneumoniae* R6 [WP_000727012.1]. fGlobal distance test (GDT) was 49% and P-value 1.9x10^{-5}, that suggest at least a good partial prediction. SWISS-MODEL (https://swissmodel.expasy.org/) (Waterhouse et al., 2018) was also used for P40 using PcsB from *Streptococcus pneumoniae* and RipA from *Mycobacterim tuberculosis* as templates (PDB nos. 4cgk.1.A and 6ewy.1.A) (35% and 28% similarity) and the general configuration was similar to that found with RaptorX2. In the case of P75, the highest P-value and GDT were found with templates 3pvqA (p= 2.6x10^{9}, GDT= 38%), 6bimA (p= 3.4x10^{9}, GDT=

36%) and 3h41A (p= $2.8x10^8$, GDT= 35%), which are respectively the NlpC/P60 family protein of *Bacteroides thetaiotaomicron* [WP_011107705.1], C40-NlpC/P60 superfamily cysteine peptidase from *Trichomonas vaginalis* G3 [XP_001276902.1] and the NlpC/P60 family protein of *Bacillus cereus* [WP_061182922.1]. This could be interpreted as suboptimal structural predictions, that again are due to the lack of more similar references.

Figure S5.- Lytic activity assay of P40 and P75 against *L.casei, E. faecalis, L. monocytogenes* and *S. aureus.* (A) pH optimization. (B) Effect of the proteins and Triton after 2 hours of incubation at the selected pH.

Figure S6.- Western blot obtained using anti-P40 polyclonal antibodies isolated form purified native P40 (Yan et al., 2007).

2. Supplementary Tables

3. **Table S1.-** List of putative proteins containing C-terminal NlpC/P60 domain in the annotated genomes of representative strains of *Lactobacillus* and *Lactococcus*, as an illustration of their abundance.

**Species_Strain**	**Ref. Genome annotation**	**Accession numbers - Annotated function**
Lactobacillus casei BL23	(Maze et al., 2010)	WP_003572828.1 - CHAP domain-containing protein[P40]<br>WP_012490875.1 - NlpC/P60 family protein[P75]<br>WP_012491782.1 - hypothetical protein<br>WP_012491062.1 - hydrolase
Lactobacillus rhamnosus GG	(Kankainen et al., 2009)	AXI93082.1 CHAP - domain-containing protein[P40]<br>AXI93342.1- glycoside hydrolase [P75]<br>AXI94896.1 - hypothetical protein DU507_10550
Lactococcus lactis subsp cremoris MG1363	(Wegmann et al., 2007;Linares et al., 2010)	AAA25230- secreted protein<br>CAL97110 - conserved hypothetical protein<br>CAC93672 - gamma-glutamyl-diamino acid-endopeptidase
Lactobacillus plantarum WCFS1	(Siezen et al., 2012)	WP_060677571.1 - NlpC/P60 family protein<br>WP_011101812.1 - NlpC/P60 family protein<br>WP_011101674.1- peptidoglycan endopeptidase<br>WP_011102154.1 - LysM peptidoglycan-binding domain-containing protein<br>WP_011101331.1 - gamma-D-glutamate-meso-diaminopimelate muropeptidase
Lactobacillus delbrueckii subsp. bulgaricus ATCC BAA-365	(van de Guchte et al., 2006)	ABJ59268.1 -Cell wall-associated hydrolase<br>ABJ57819.1 - Cell wall-associated hydrolase<br>ABJ59266.1 - Cell wall-associated hydrolase<br>ABJ59129.1 - dipeptidyl-peptidase VI, Cysteine peptidase, MEROPS family C40<br>ABJ59265.1 - Cell wall-associated hydrolase
Lactobacillus brevis ATCC 367	(Makarova et al., 2006)	ABJ64517.1 - Cell wall-associated hydrolase<br>ABJ63531.1 - D-alanyl-D-alanine carboxypeptidase<br>ABJ64515.1 - Cell wall-associated hydrolase
Lactobacillus gasseri ATCC 33323	(Makarova et al., 2006)	ABJ60987.1 - Cell wall-associated hydrolase

Table S2.- FASTA sequences of BL23 *cmu*B gene and *L. paracasei* variants of minisatellites.

```
>NC_010999.1:277055-278539 Lactobacillus casei BL23 complete genome, strain BL23
> ref|NC_010999.1
ATGGTAGATGCAAAGAAAGTATTGTCAGTTACGGCAGGATTCGTTGGTGCTGCCGGTTTGGCAACTTTAG
CTACCGGCGCTAATACTGTTTCCGCTTCAACGGGGACAGTCAATTACAAATCGGGTGCGACCACCGTATG
GAATAGTCCATCATGGCATCAAGTAAAACGCTATGTGACTTTTGGTGACAAAGTACAAGTTCTAGGTCAA
AAAGTTGACCGAAACGGTGCTACTTGGTATAAAGTCGGCGACAATCAGTGGATCCCTCTCATTTATTTGA
ATTTTGACGGCAAGACTGTCACCGTTCAAGCACCAGAACAAACTGCCAGTCAGGCACCGGTTAGCCAAGC
GCCAGCTAGCCAAGCACCTGCAAGTCAGGCACCTGCGAGTCAAGCAGCCGCACAGCCTGATACCCAGACA
GCAAACATTCAGTTATATGTTAAAAACATTGGTTCTGCAGTCACAGTTTGGGCAACACCAGCGTACAGTC
AGGCAACTGGCCAGTATCTGGAAGGCAACCAGACGTTGACGGCGGTTGCCCAGTTGCAAGCCAATGGCGA
AACATGGTATCGGTTAGCAAATGGCGGTTACGTGCCAGAACGTTTTGCTAGCACAACACCTGCACCAGCA
CCGCAGTCATCTGCTGCAACGAGTGTTGCGGCACCGACTGCGCCTGTATCTGATGCAACGGCTTCGAACG
CAGCTGCTTCAAATGCCGCCGCTTCGGATGTTGCTGTCTCCAGTGCTGCAGCGTCTAGTGCCGCGGCTTC
ATTAGCTGCCGCTTCAGAAGCTGCTGCGGTTGCGAATGCTTCTAGCGCTGCTGCATCTTCTGCTGCCGCT
GTAGCGAGTGCTGCCGCAGAATCCAGTGCTGCTGCAGAGTCCAGTGCTGCCGCATCGAAGGCTGCTGCTG
ATTCTAGCGCTGCAGCTGTGCAGACTACGACGCCTGAGTCCAGTGCTGCGCCTGCAACGACACAGGTTGA
TGCAACTCAGGAACAGCAACAGCAGGCAGAACCAAGCAATACGGTCAATACCGAGGAAACGACAAATAAC
GCGACGCCGACTCCTGCACCAACGCCGACGCCTGCACCAACCCCAGCCCCAGCTCCTGTTACGCCGTCAC
GTCAGGCAAAAATTCAGGCGGTTATCTCAATTGCTGAACAGCAAATTGGGAAACCTTATGTATGGGGTGG
CAAGGGTCCTAACAGCTTTGACTGCTCAGGCTTGATGTATTATGCCTTTTTGAACGGCGCCGGTGTTAAC
ATCGGTGGTTGGACAGTGCCACAAGAATCTTCTGGTCAGCAAGTCTCACTGAGTGCACTTCAGCCTGGTG
ATTTGCTCTTCTGGGGCGGACATGGTAGCTCTTACCACGTAGCGCTCTACATTGGTGGCGGTACAATGAT
TCAGGCACCACAACCAGGTGAAAATGTGAAGTACACGGCATTAGCTTACTTCATGCCTGATTTTGCTGTT
CGTCCTTCACTATAA
```

Lactobacillus paracasei strain Zhang, complete genome

```
     gene            294624..296105
                     /locus_tag="LCAZH_0300"
     CDS             294624..296105
                     /locus_tag="LCAZH_0300"
                     /codon_start=1
                     /transl_table=11
                     /product="cell wall-associated hydrolase"
                     /protein_id="ADK17593.1"
                     /translation="MVDAKKVLSVTAGFVGAAGLATLATGANTVSASTGTVNYKSGAT
                     TVWNSPSWHQVKRYVTFGDKVQVLGQKVDRNGATWYKVGDNQWIPGIYLNFDGKTVTV
                     QAPEQTASQAPVSQAPASQAPASQAPASQAAAQPDTQTATIQLYVKNIGSAVTVWATP
                     AYSQATGQYLEGNQTLTAVAQLQANGETWYRLANGGYVPERFASTTPAPAPQSSAATS
                     VAAPTAPVSDATASNAAASDAAASSAAASSAAASLAVASEAAASVANASSAAASSAAV
                     ASAAAESSTAAESSAAAESSAAASKAAADSSAAAVQTTTPESSAAPATTQVDATQEQQ
                     QQAEPSITVNAEETTNNATPTPAPTPTPAPTPAPVTPSRQAKIQAVISIAEQQIGKPY
                     VWGGKGPNSFDCSGLMYYAFLNGAGVNIGGWTVPQESSGQQVSLSALQPGDLLFWGGH
                     GSSYHVALYIGGGTMIQAPQPGENVKYTALAYFMPDFAVRPSL"
```

```
>CP001084.2:294624-296105 Lactobacillus paracasei strain Zhang, complete genome
ATGGTAGATGCAAAGAAAGTATTGTCAGTTACGGCAGGATTCGTTGGTGCTGCCGGTTTGGCAACTTTAG
CTACCGGCGCTAATACTGTTTCCGCTTCAACGGGGACAGTCAATTACAAATCGGGTGCGACCACCGTATG
GAATAGTCCATCATGGCATCAAGTAAAACGCTATGTGACTTTTGGTGACAAAGTACAAGTTCTAGGTCAA
AAAGTTGACCGAAACGGTGCTACTTGGTATAAAGTCGGCGACAATCAGTGGATCCCTGGCATTTATTTGA
ATTTTGACGGCAAGACTGTCACCGTTCAAGCACCAGAACAAACTGCCAGTCAGGCACCGGTTAGCCAAGC
GCCAGCTAGCCAAGCACCTGCAAGTCAGGCACCTGCGAGTCAAGCAGCCGCACAGCCTGATACCCAGACA
GCAACCATTCAGTTATATGTTAAAAACATTGGTTCTGCAGTCACCGTTTGGGCAACACCAGCGTACAGTC
AGGCAACTGGCCAGTATCTGGAAGGCAACCAGACGTTGACGGCGGTTGCCCAGTTGCAAGCCAATGGCGA
AACATGGTATCGGTTAGCAAATGGCGGTTACGTGCCAGAACGTTTTGCTAGCACAACACCTGCACCAGCA
CCGCAGTCATCTGCTGCAACGAGTGTTGCGGCACCGACTGCGCCTGTATCTGATGCAACGGCTTCGAACG
CAGCTGCTTCGGATGCTGCTGCCTCCAGTGCCGCAGCGTCCAGTGCTGCAGCTTCATTGGCTGTCGCTTC
AGAAGCTGCTGCTTCGGTTGCAAATGCCTCTAGCGCTGCTGCATCGTCTGCTGCTGTAGCGAGTGCTGCC
GCAGAATCCAGTACGGCTGCAGAATCCAGTGCTGCTGCAGAATCCAGTGCTGCTGCATCTAAGGCTGCTG
CTGATTCTAGCGCTGCAGCTGTGCAGACTACGACGCCTGAGTCCAGTGCTGCGCCTGCAACGACACAGGT
TGATGCAACTCAGGAACAGCAACAGCAGGCAGAACCAAGCATTACGGTCAATGCCGAGGAAACGACAAAT
AACGCGACGCCGACTCCTGCACCAACGCCGACGCCTGCACCAACCCCAGCCCCTGTTACGCCGTCACGTC
AGGCAAAAATTCAGGCGGTTATCTCAATTGCTGAACAGCAAATTGGGAAACCTTATGTATGGGGTGGCAA
GGGTCCTAACAGCTTTGACTGCTCAGGCTTGATGTATTATGCCTTTTTGAACGGCGCCGGTGTTAACATC
GGTGGTTGGACAGTGCCACAAGAATCTTCTGGTCAGCAAGTCTCACTGAGTGCACTTCAGCCTGGTGATT
TGCTCTTCTGGGGCGGACATGGTAGCTCTTACCACGTAGCGCTCTACATTGGTGGCGGTACAATGATTCA
GGCACCACAACCAGGTGAAAATGTGAAGTATACGGCATTAGCTTACTTCATGCCTGATTTTGCTGTTCGT
CCTTCACTATAA
```

```
CP016355.1 Lactobacillus paracasei subsp. paracasei strain TMW 1.1434:
gene            307325..308785
                     /locus_tag="BBD24_01395"
     CDS             307325..308785
                     /locus_tag="BBD24_01395"
                     /inference="EXISTENCE: similar to AA
                     sequence:RefSeq:WP_016363833.1"
                     /note="Derived by automated computational analysis using
                     gene prediction method: Protein Homology."
                     /codon_start=1
                     /transl_table=11
                     /product="glycoside hydrolase"
                     /protein_id="AUB99713.1"
                     /translation="MVDAKKVLSVTAGFVGAAGLATLATGANTVSASTGTVNYKSGAT
                     TVWNSPSWHQVKRYVTFGDKVQVLGQKVDRNGATWYKVGDNQWIPGIYLNFDGKTVTV
                     QAPEQTASQAPVSQAPASQAPASQAPASQAAAQPDTQTANIQLYVKNIGSAVTVWATP
                     AYSQATGQYLEGNQTLTAVAQLQANGETWYRLANGGYVPERFASTTPAPAPQSSAATS
                     VAAPTAPVSDATASNAAASNAAASDAAVSSAAASSAAASLAAASLAAAVANASSAAAS
                     SAAAVASAAAESSAAAESSAAASKAAADSSAAAVQTTTPESSAAPATTQVDATQEQQQ
                     QAEPSNTVNAEETTNNATPAPTPAPAPVTPSRQAKIQAVISIAEQQIGKPYVWGGKGP
                     NSFDCSGLMYYAFLNGAGVNIGGWTVPQESSGQQVSLSALQPGDLLFWGGRGSSYHVA
                     LYIGGGTMIQAPQPGENVKYTALAYFMPDFAVRPSL"
```

```
>CP016355.1:307325-308785 Lactobacillus paracasei subsp. paracasei strain TMW 1.1434 chromosome,
complete genome
ATGGTAGATGCAAAGAAAGTATTGTCAGTTACGGCAGGATTCGTTGGTGCTGCCGGTTTGGCAACTTTAG
CTACCGGCGCTAATACTGTTTCCGCTTCAACGGGGACAGTCAATTACAAATCGGGTGCGACCACCGTATG
GAATAGTCCATCATGGCATCAAGTAAAACGCTATGTGACTTTTGGTGACAAAGTACAAGTTCTAGGTCAA
AAAGTTGACCGAAACGGTGCTACTTGGTATAAAGTCGGCGACAATCAGTGGATCCCTGGCATTTATTTGA
ATTTTGACGGCAAGACTGTCACCGTTCAAGCACCAGAACAAACTGCCAGTCAGGCACCGGTTAGCCAAGC
GCCAGCTAGCCAAGCACCTGCAAGTCAGGCACCTGCGAGTCAAGCAGCCGCACAGCCTGATACCCAGACA
GCAAACATTCAGTTATATGTTAAAAACATTGGTTCTGCAGTCACAGTTTGGGCAACACCAGCGTACAGTC
AGGCAACTGGCCAGTATCTGGAAGGCAACCAGACGTTGACGGCGGTTGCCCAGTTGCAAGCCAATGGCGA
AACATGGTATCGGTTAGCAAATGGCGGTTACGTGCCAGAACGTTTTGCTAGCACAACACCTGCACCAGCA
CCGCAGTCATCTGCTGCAACGAGTGTTGCGGCACCGACTGCGCCTGTATCTGATGCAACGGCTTCGAACG
CAGCTGCTTCAAATGCCGCCGCTTCGGATGCTGCTGTCTCCAGTGCTGCAGCGTCTAGTGCCGCGGCTTC
ATTAGCTGCCGCTTCATTAGCTGCTGCGGTTGCGAATGCTTCTAGCGCTGCTGCATCTTCTGCTGCCGCT
GTAGCGAGTGCTGCCGCAGAATCCAGTGCTGCTGCAGAATCTAGTGCTGCTGCATCAAAGGCTGCTGCTG
ATTCTAGCGCTGCAGCTGTGCAGACTACGACGCCTGAGTCCAGTGCTGCGCCTGCAACGACACAGGTTGA
TGCAACTCAGGAACAGCAACAGCAGGCAGAACCAAGCAATACGGTCAATGCCGAGGAAACGACAAATAAC
GCGACGCCTGCACCAACCCCAGCCCCAGCTCCTGTTACGCCGTCACGTCAGGCAAAAATTCAGGCAGTTA
TCTCAATTGCTGAACAGCAAATTGGGAAACCTTATGTATGGGGTGGCAAGGGTCCTAACAGCTTTGACTG
CTCAGGCTTGATGTATTATGCCTTTTTGAACGGCGCCGGTGTTAACATCGGTGGTTGGACAGTGCCACAA
GAATCTTCTGGTCAGCAAGTCTCACTGAGTGCACTTCAGCCTGGTGATTTGCTCTTCTGGGGCGGACGTG
GTAGCTCTTACCACGTAGCGCTCTACATTGGTGGCGGTACAATGATTCAGGCACCACAACCAGGTGAAAA
TGTGAAGTATACGGCATTAGCTTACTTCATGCCTGATTTTGCTGTTCGTCCTTCACTATAA
```

```
AP012541.1 Lactobacillus paracasei subsp. paracasei JCM 8130
gene            275403..276896
                     /locus_tag="LBPC_0264"
     CDS             275403..276896
                     /locus_tag="LBPC_0264"
                     /codon_start=1
                     /transl_table=11
                     /product="putative cell wall-associated hydrolase"
                     /protein_id="BAN70560.1"
                     /translation="MVDAKKVLSVTAGFVGAAGLATLATGANTVSASTGTVNYKSGAT
                     TVWNSPSWHQVKRYVTFGDKVQVLGQKVDRNGATWYKVGDNQWIPGIYLNFDGKTVTV
                     QAPEQTASQAPVSQAPASQAPASQAPASQAATQPDTQTANIQLYVKNIGSAVTVWATP
                     AYSQATGQYLEGNQTLTAVAQLQANGETWYRLANGGYVPERFASTTPAPAPQSSAATS
                     VAAPTAPVSDATASNAAASNAAASDAAVSSAAASSAAASSAAASSAAASSAAASSAAA
                     SLAVASEAAASVANASSAAASSAADSSAAAVQTTTPESSAAPATTQVDATQEQQQQAE
                     PSNTVNAEETTNNATPTPAPTPTPAPTPTPAPTPAPAPVTPSRQAKIQAVISIAEQQI
                     GKPYVWGGKGPNSFDCSGLMYYAFLNGAGVNIGGWTVPQESSGQQVSLSALQPGDLLF
                     WGGHGSSYHVALYIGGGTMIQAPQPGENVKYTALAYFMPDFAVRPSL"
```

```
>AP012541.1:275403-276896 Lactobacillus paracasei subsp. paracasei JCM 8130 DNA, complete genome
ATGGTAGATGCAAAGAAAGTATTGTCAGTTACGGCAGGATTCGTTGGTGCTGCCGGTTTGGCAACTTTAG
CTACCGGCGCTAATACTGTTTCCGCTTCAACGGGGACAGTCAATTACAAATCGGGTGCGACCACCGTATG
GAATAGTCCATCATGGCATCAAGTAAAACGCTATGTGACTTTTGGTGACAAAGTACAAGTTCTAGGTCAA
```

```
AAAGTTGACCGAAACGGTGCTACTTGGTATAAAGTCGGCGACAATCAGTGGATCCCTGGCATTTATTTGA
ATTTTGACGGCAAGACTGTCACCGTTCAAGCACCAGAACAAACTGCCAGTCAGGCACCGGTTAGCCAAGC
GCCAGCTAGCCAAGCACCTGCAAGTCAGGCACCTGCGAGTCAAGCAGCCACACAGCCTGATACCCAGACA
GCAAACATTCAGTTATATGTTAAAAACATTGGTTCTGCAGTCACAGTTTGGGCAACACCAGCGTACAGTC
AGGCAACTGGCCAGTATCTGGAAGGCAACCAGACGTTGACGGCGGTTGCCCAGTTGCAAGCCAATGGCGA
AACATGGTATCGGTTAGCAAATGGCGGTTACGTGCCAGAACGTTTTGCTAGCACAACACCTGCACCAGCA
CCGCAGTCATCTGCTGCAACGAGTGTTGCGGCACCGACTGCGCCTGTATCTGATGCAACGGCTTCGAACG
CAGCTGCTTCAAATGCCGCCGCTTCGGATGCTGCTGTCTCCAGTGCTGCAGCGTCCAGTGCCGCAGCGTC
CAGTGCTGCAGCGTCCAGTGCCGCAGCGTCCAGTGCCGCAGCGTCCAGTGCCGCAGCGTCATTGGCTGTC
GCTTCAGAAGCTGCTGCTTCGGTTGCAAATGCCTCTAGCGCTGCTGCATCGTCTGCTGCTGATTCTAGCG
CTGCAGCTGTGCAGACTACGACGCCTGAGTCCAGTGCTGCGCCTGCAACGACACAGGTTGATGCAACTCA
GGAACAGCAACAGCAGGCAGAACCAAGCAATACGGTCAATGCCGAGGAAACGACAAATAACGCGACGCCG
ACTCCTGCACCAACGCCGACTCCTGCACCAACGCCGACTCCTGCACCAACCCCAGCCCCAGCTCCTGTTA
CGCCGTCACGTCAGGCAAAAATTCAGGCGGTTATCTCAATTGCTGAACAGCAAATTGGGAAACCTTATGT
ATGGGGTGGCAAGGGTCCTAACAGCTTTGACTGCTCAGGCTTGATGTATTATGCCTTTTTGAACGGCGCC
GGTGTTAACATCGGTGGTTGGACAGTGCCACAAGAATCTTCTGGTCAGCAAGTCTCACTGAGTGCACTTC
AGCCTGGTGATTTGCTCTTCTGGGGCGGACATGGTAGCTCTTACCACGTAGCGCTCTACATTGGTGGCGG
TACAATGATTCAGGCACCACAACCAGGTGAAAATGTGAAGTATACGGCATTAGCTTACTTCATGCCTGAT
TTTGCTGTTCGTCCTTCACTATAA
```

Tables S3.- Sequences used in the phylogenetic analysis.

Table S3.1.- P40 homologous sequences used for phylogenetic reconstructions.

**Strain**	**Acc. Nº**
Lactobacillus casei BL23	CAQ65155
Lactobacillus paracasei DUP 13076	WP_101869740
Lactobacillus paracasei L9D	WP_071252149
Lactobacillus paracasei subsp. *paracasei* Lpp221	WP_016379272
Lactobacillus casei subsp. *casei* ATCC 393	WP_025013825
Lactobacillus rhamnosus HN001	WP_005687968
Lactobacillus rhamnosus GG	WP_014568889
Lactobacillus zeae DSM 20178	WP_010490240
Lactobacillus sakei subsp. *sakei* 23K 1	WP_011374356
Lactobacillus sakei subsp. *sakei* 23K 2	WP_011373952
Lactobacillus murinus DSM 20452	WP_004051788
Lactobacillus animalis DSM 20602	KRM59177
Lactobacillus curvatus MRS6	WP_089557112
Lactobacillus graminis DSM 20719	WP_057908021
Lactobacillus fuchuensis DSM 14340	WP_056950269
Lactobacillus manihotivorans DSM 13343	WP_056964416
Lactobacillus nasuensis JCM 17158	WP_054723415
Enterococcus asini ATCC 700915	WP_010752994
Lactococcus lactis subsp. *lactis* K337	WP_058204561
Lactococcus piscium DSM 6634	WP_096813813
Streptococcus equi subsp. *ruminatorum* CECT 5772	WP_037582442
Streptococcus pneumoniae R6	WP_000727012
Streptococcus sanguinis 2908	CEL91468
Vagococcus teuberi DSM 21459	WP_071456026

Table S3.2.- P75 homologous sequences used for phylogenetic reconstructions.

**Strain**	**Acc. Nº**
Lactobacillus casei BL23	CAQ65403
Lactobacillus paracasei lpp122 1	WP_016383801
Lactobacillus paracasei lpp122 2	WP_016383443
Lactobacillus rhamnosus GG	WP_005687638

Lactobacillus casei subsp. *casei* ATCC 393	WP_025013844
Lactobacillus casei LC5	WP_087911410
Lactobacillus zeae DSM 20178	WP_010492712
Lactobacillus nasuensis JCM 17158	WP_054723265
Lactobacillus cameliae DSM 22697	WP_056989569
Lactobacillus fuchuensis DSM 14340	WP_056950327
Lactobacillus sakei subsp. *sakei* 23K	WP_011374023
Lactobacillus sakei FAM18311	WP_076632543
Lactobacillus curvatus FBA2	WP_064777492
Lactobacillus graminis DSM 20719	WP_057907367
Lactobacillus amylophilus DSM 20533	WP_056947108
Lactobacillus helsingborgensis Bma 5	WP_046327598
Lactobacillus manihotivorans DSM 13343	WP_082611783
Lactobacillus perolens DSM 12744	WP_083487735
Lactobacillus shenzhenensis LY-73	WP_081701288
Lactobacillus harbinensis DSM 16991	WP_027829605
Lactobacillus harbinensis BM-LB13908	WP_063516512
Lactobacillus farciminis DSM 111452	WP_059074409
Lactobacillus farciminis DSM 20184	WP_010020650
Lactobacillus futsaii JCM 17355	WP_057811975
Lactobacillus kimchiensis DSM 24716	WP_057881517
Lactobacillus versmoldensis DSM 14857	WP_010624150
Lactobacillus ginsenosidimutans EMML 3041	WP_048704780
Lactobacillus nodensis DSM 19682	WP_056979871
Lactobacillus nantensis DSM 16982	WP_057891330
Lactobacillus heilongjiangensis DSM 28069	WP_041499677

Table S4.- Blast alignment of *L.casei/paracasei/rhamnosus* genes encoding P40 proteins for the selection of PCR primers

```
Database: Nucleotide collection (nt)
           36,527,618 sequences; 117,866,738,766 total letters
Query= gi|191636824:c23411-22176 Lactobacillus casei BL23 complete genome,
strain BL23

Length=1236
                                                                 Score     E
Sequences producing significant alignments:                       (Bits)  Value

emb|HE970764.1|  Lactobacillus casei W56 complete genome           2283     0.0
gb|CP002618.1|  Lactobacillus casei BD-II, complete genome         2283     0.0
gb|CP002616.1|  Lactobacillus casei LC2W, complete genome          2283     0.0
emb|FM177140.1|  Lactobacillus casei BL23 complete genome, str...  2283     0.0
gb|CP006690.1|  Lactobacillus casei 12A, complete genome           2067     0.0
gb|CP001084.2|  Lactobacillus casei str. Zhang, complete genome    2061     0.0
gb|CP005486.1|  Lactobacillus casei LOCK919, complete genome       2061     0.0
gb|CP005484.1|  Lactobacillus rhamnosus LOCK900, complete genome   900      0.0
dbj|AP012544.1|  Lactobacillus casei subsp. casei ATCC 393 DNA...  889      0.0
dbj|AP011548.1|  Lactobacillus rhamnosus ATCC 53103 DNA, compl...  883      0.0
emb|FM179322.1|  Lactobacillus rhamnosus GG whole genome seque...  883      0.0

OLIGONUCLEOTIDES:
LcasP40N for 5’- GTTGGATCCGATACAAGCGACAG (BAmHI
LcasP40N rev 5’-GTAGGGCCCTTATTGCTTATTCAAAGC (SmaI)GCTTTGAATAAGC
LcasP40C for 5’- GTTGGATCCGCTGATCATAAAGATG
(BamHI)
LcasP40C rev 5’-GTAGGGCCCTTACCGGTGGATATAA (SmaI)
TTATATCCACCGGTAA
```

For the L.rhamnosus sequences, see at the end full sequences
RhamP40N for 5’- GTTGGATCCGACACAAGTGCCAG (BamHI)
RhamP40N rev 5’-GTAGGGCCCTTATTCTTTGTTCAAAGC (SmaI)
GCTTTGAACAAAGAA
RhamP40C for 5’- GTTGGATCCGCTGATCACAAAGAC
(BamHI)
RhamP40C rev 5’-GTAGGGCCCTTACCGGTGGATGTAA (SmaI)
TTACATCCACCGGT

ALIGNMENTS

```
Query     1      ATGAAATTCAATAAAGTCATGATCACGTTGGTTGCTGCAGTTACCTTAGCAGGTTCTGCT  60
HE970764  23408  ............................................................  23349
CP002618  23409  ............................................................  23350
CP002616  23409  ............................................................  23350
FM177140  23411  ............................................................  23352
CP006690  22250  ............................................................  22191
CP001084  22759  ............................................................  22700
CP005486  20930  ............................................................  20871
CP005484  25290  ................CA.....G..A........A..............G.......T.  25231
AP012544  20379  ................CT.....T..A........C.......................C  20438
AP011548  31251  ................CA.....G..A........A..............G.......T.  31192
FM179322  31251  ................CA.....G..A........A..............G.......T.  31192

Query     61     AGCGCCGTAACACCAGTTTTCGCTGATACAAGCGACAGTATCGCTTCCAACAAAAGCGAA  120
HE970764  23348  ............................................................  23289
CP002618  23349  ............................................................  23290
CP002616  23349  ............................................................  23290
FM177140  23351  ............................................................  23292
CP006690  22190  ............................................................  22131
CP001084  22699  ............................................................  22640
CP005486  20870  ............................................................  20811
CP005484  25230  ..............G...........C.....T.C...C.....A..T.....G......  25171
AP012544  20439  ..............G...........C.....T.C...C........T............  20498
AP011548  31191  ......T.......G...........C.....T.C...C.....A..T.....G......  31132
FM179322  31191  ......T.......G...........C.....T.C...C.....A..T.....G......  31132

Query     121    ACCAATGCACTGTTGAAGCAAATTGAAGATGCGAACACAGAAGTTATCAATTTGAACAAG  180
HE970764  23288  ............................................................  23229
CP002618  23289  ............................................................  23230
CP002616  23289  ............................................................  23230
FM177140  23291  ............................................................  23232
CP006690  22130  ............................................................  22071
CP001084  22639  ............................................................  22580
CP005486  20810  ............................................................  20751
CP005484  25170  .....C.ATT.A..A........C....CA..T.....T.....G.....CC.C......  25111
AP012544  20499  ..G..C.ATT.A..A..A.....C....CA..C.....T..G..C......C.T.....A  20558
AP011548  31131  .....C.ATT.A..A........C....CA..T.....T.....G.....CC.C.....A  31072
FM179322  31131  .....C.ATT.A..A........C....CA..T.....T.....G.....CC.C.....A  31072

Query     181    CAGATCGATACCAAGAATGGTCAAATCAGCGATGCAACGGCTAAGATCAGCGCAACTGAT  240
HE970764  23228  ............................................................  23169
CP002618  23229  ............................................................  23170
CP002616  23229  ............................................................  23170
FM177140  23231  ............................................................  23172
CP006690  22070  ............................................................  22011
CP001084  22579  ............................................................  22520
CP005486  20750  ............................................................  20691
CP005484  25110  .....T...G.T........CG.......T..C..T..T..C........T.....G...  25051
AP012544  20559  .....T..CGAT......TCG........T.....C..A..C........T.....C...  20618
AP011548  31071  .....T...G.T........CG.......T..C..C..T..C........T.....G...  31012
FM179322  31071  .....T...G.T........CG.......T..C..C..T..C........T.....G...  31012

Query     241    GCCAAGATTTCATCACTCAGCGGTGAAATTGTTGCCGCTCAAAAGAATGTCACAGCACGC  300
HE970764  23168  ............................................................  23109
CP002618  23169  ............................................................  23110
CP002616  23169  ............................................................  23110
FM177140  23171  ............................................................  23112
CP006690  22010  ............................................................  21951
CP001084  22519  ............................................................  22460
CP005486  20690  ............................................................  20631
CP005484  25050  ........CG....GT.G..T..C......ACC..T...........C...G....C..G  24991
```

AP012544	20619	...C....CAA....T.G.....CC....CACG..T..C........C..G..G.....G	20678
AP011548	31011	........CG....GT.G..T..C.....CACC..T...........C...G....C..G	30952
FM179322	31011	........CG....GT.G..T..C.....CACC..T...........C...G....C..G	30952

Query	301	AA**G**AACAACTTGAAAGATCAGTTGATCTCCTTGCaaaaaaaGGCTGGCAACTCTGTCAGC	360
HE970764	23108	....	23049
CP002618	23109	....	23050
CP002616	23109	....	23050
FM177140	23111	....	23052
CP006690	21950	..A...	21891
CP001084	22459	..A...	22400
CP005486	20630	..A...	20571
CP005484	24990	..............G.....A.....T...C.T.....G..A..C....GT..A..T...	24931
AP012544	20679	.................C..AC....T...C.T.....G..........G...G..T...	20738
AP011548	30951	..............G.....A.....T...C.T.....G..A..C....GT..A..T...	30892
FM179322	30951	..............G.....A.....T...C.T.....G..A..C....GT..A..T...	30892

Query	361	GGCAATGTCTATATCGACTTTGTTCTGAATTCCCAGAA-CT-TGTCTGACTTAATTGCCC	418
HE970764	23048	......................................-..-..................	22991
CP002618	23049	......................................-..-..................	22992
CP002616	23049	......................................-..-..................	22992
FM177140	23051	......................................-..-..................	22994
CP006690	21890	......................................-..-..................	21833
CP001084	22399	......................................-..-..................	22342
CP005486	20570	......................................-..-..................	20513
CP005484	24930	..............T..T..C..GT....C..A..-..-G.C.A........G.......	24873
AP012544	20739	...........C........C..GT....C..A..A.GT..-.-..G.....G..C....	20796
AP011548	30891	..............T..T..C..GT....C..A..-..-G.C.A........G.......	30834
FM179322	30891	..............T..T..C..GT....C..A..-..-G.C.A........G.......	30834

Query	419	GCACCATGACGGTTGGCAAACTTAGCCAAGCCAGCAAAGATGCGC-TT-GATGCCGTGAC	476
HE970764	22990	....-..-...........	22933
CP002618	22991	....-..-...........	22934
CP002616	22991	....-..-...........	22934
FM177140	22993	....-..-...........	22936
CP006690	21832	....-..-...........	21775
CP001084	22341	....-..-...........	22284
CP005486	20512	....-..-...........	20455
CP005484	24872	.T........A..C.....GT.A..T..............--...T..G.....G..A..	24815
AP012544	20797	..........C...........C..............G..A....-.G-..C..T.....	20854
AP011548	30833	.T........A..C.....GT.A..T..............--...T..G.....G..A..	30776
FM179322	30833	.T........A..C.....GT.A..T..............--...T..G.....G..A..	30776

Query	477	AGTTGCCAAGGATAAATTAGCTGGCTTGAAGACGGAACAGGAAAATGCCCGTCAGACGCT	536
HE970764	22932	....	22873
CP002618	22933	....	22874
CP002616	22933	....	22874
FM177140	22935	....	22876
CP006690	21774	....	21715
CP001084	22283	....	22224
CP005486	20454	....	20395
CP005484	24814	C..C.....A.....G.....C.CT.......GT..........CG..A........CT.	24755
AP012544	20855	C...........C...C.G..A..AC......ACC.........CA..G.........T.	20914
AP011548	30775	C..C.....A.....G.....C.CT.......GT..........CG..A........CT.	30716
FM179322	30775	C..C.....A.....G.....C.CT.......GT..........CG..A........CT.	30716

Query	537	GGTTTCAACTAAGGC-T-TCATTGGAAACCCAGAAATCACAGTTGGTTTCCCTACAAAAA	594
HE970764	22872	...............-.-..	22815
CP002618	22873	...............-.-..	22816
CP002616	22873	...............-.-..	22816
FM177140	22875	...............-.-..	22818
CP006690	21714	...............-.-..	21657
CP001084	22223	...............-.-..	22166
CP005486	20394	...............-.-..	20337
CP005484	24754	......G.....A..A.C..-..-.....G..A..G......C.A.AGA....T.....-	24698
AP012544	20915	.........C...T.-A-..GC.T...........G..T.........A..T.G.....-	20971
AP011548	30715	......G.....A..A.C..-..-.....G..A..G......C.A.AGA....T.....-	30659
FM179322	30715	......G.....A..A.C..-..-.....G..A..G......C.A.AGA....T.....-	30659

Query	595	GA-TGCTAACGATAAACAAGATGC**T**TTGAATAAGCAAATTGCTGATCATAAAGATGAGTT	653
HE970764	22814	..-...	22756
CP002618	22815	..-...	22757
CP002616	22815	..-...	22757

```
FM177140  22817  ..-.........................................................  22759
CP006690  21656  ..-..............................A..........................  21598
CP001084  22165  ..-..............................A..........................  22107
CP005486  20336  ..-..............................A..........................  20278
CP005484  24697  ..CC..A.G......G..............C..AG.......C.....C.....C..A..  24638
AP012544  20972  ..CA....G...C..G........C.....C........CAA...C..CCGT..C..A..  21031
AP011548  30658  ..CC..A.G......G..............C..AG.............C.....C..A..  30599
FM179322  30658  ..CC..A.G......G..............C..AG.............C.....C..A..  30599

Query     654    GGTTGCTTTGCAAAGCCAATTTGCTCAAGAACAGTCTGAAG-CAGCTGCTGCAACCCAAG  712
HE970764  22755  .........................................-..................  22697
CP002618  22756  .........................................-..................  22698
CP002616  22756  .........................................-..................  22698
FM177140  22758  .........................................-..................  22700
CP006690  21597  ........................C.............C..-..................  21539
CP001084  22106  ........................C.............C..-..................  22048
CP005486  20277  ........................C.............C..-..................  20219
CP005484  24637  ......AC.C.....T.................A..A....-....CAAA........G.  24579
AP012544  21032  A.................G..C..A..G......-GAA...A.C.....G........G.  21090
AP011548  30598  ......AC.C.....T.................A..A....-....CAAA........G.  30540
FM179322  30598  ......AC.C.....T.................A..A....-....CAAA........G.  30540

Query     713    CCGCTTTGAAGACCGTTGCCGCTTCAAC--C-GCAAGTTCTTCTA-C-----T------A  757
HE970764  22696  ............................--.-.............-.-----.------.  22652
CP002618  22697  ............................--.-.............-.-----.------.  22653
CP002616  22697  ............................--.-.............-.-----.------.  22653
FM177140  22699  ............................--.-.............-.-----.------.  22655
CP006690  21538  ............................--.-.............-.-----.------.  21494
CP001084  22047  ............................--.-.............-.-----.------.  22003
CP005486  20218  ............................--.-.............-.-----.------.  20174
CP005484  24578  .T..C........A.CG..T..A.....TG.TT........A...G.-----.C-----.  24529
AP012544  21091  .T...........G..C..T..A.....--.-.GTTC.G.A..A.G.AAGAG.GCCGAA.  21147
AP011548  30539  .T..C........A.CA..T..A.....TG.TT........A.C.G.-----.CA----.  30489
FM179322  30539  .T..C........A.CA..T..A.....TG.TT........A.C.G.-----.CA----.  30489

Query     758    C--C----AGCAGT-TC-A-AGC-AGCG-CAACTTCAA-CC--AGCAC-C-A-TCG---C  797
HE970764  22651  .--.----......-..-.-...-....-.........-..--.....-.-.-...---.  22612
CP002618  22652  .--.----......-..-.-...-....-.........-..--.....-.-.-...---.  22613
CP002616  22652  .--.----......-..-.-...-....-.........-..--.....-.-.-...---.  22613
FM177140  22654  .--.----......-..-.-...-....-.........-..--.....-.-.-...---.  22615
CP006690  21493  .--.----......-..-.-...-....-.........-..--.....-.-.-...---.  21454
CP001084  22002  .--T----......-..-.-...-....-.........-..--.....-.-.-...---.  21963
CP005486  20173  .--T----......-..-.-...-....-.........-..--.....-.-.-...---.  20134
CP005484  24528  A--.----TT..AA-.--.-..--..T.C....AG..G-TG--TT.T.T.G.C.G.TAC.  24484
AP012544  21148  G--.----......-..A.C.A.T.AG.-.TG......G..AA.....-.-C-..T---.  21193
AP011548  30488  .TT.AAAT.AG...GC.A.C...-..T.-TTCT...G.-.T--G.T..-.---.------  30444
FM179322  30488  .TT.AAAT.AG...GC.A.C...-..T.-TTCT...G.-.T--G.T..-.---.------  30444

Query     798    -CAACAACACATCAAGTTCTTCAAACAACAGTTCCTC--C-A-T---T-ATCAACAACAA  848
HE970764  22611  -....................................--.-.-.---.-...........  22561
CP002618  22612  -....................................--.-.-.---.-...........  22562
CP002616  22612  -....................................--.-.-.---.-...........  22562
FM177140  22614  -....................................--.-.-.---.-...........  22564
CP006690  21453  -.................................T..--T-G-.---.-....C......  21403
CP001084  21962  -.................................T..--T-G-.---.-....C......  21912
CP005486  20133  -.................................T..--T-G-.---.-....C......  20083
CP005484  24483  T..T....-..A.-.C...C..C....G.G..G....AT.T.C.G--.G....G..G...  24428
AP012544  21194  -..-....-..G.-.CC.....C....G.G..G....AT.-.-.CCG.C....G.....C  21247
AP011548  30443  -..T....-..A.-.C......C....G.G..G....AT.T.C.G--.G....G..G...  30389
FM179322  30443  -..T....-..A.-.C......C....G.G..G....AT.T.C.G--.G....G..G...  30389

Query     849    CACGACTTCAGGTAGCGGCAGTCACGCTGATTACAGCAGTTCAGGCAACACGTATCCTTG  908
HE970764  22560  ............................................................  22501
CP002618  22561  ............................................................  22502
CP002616  22561  ............................................................  22502
FM177140  22563  ............................................................  22504
CP006690  21402  ...AG.......C..T.....C........C.T...TG.......T.....C........  21343
CP001084  21911  ...AG.......C..T.....C........C.T...TG.......T.....C........  21852
CP005486  20082  ...AG.......C..T.....C........C.T...TG.......T.....C........  20023
CP005484  24427  ...TG................C..T............G.......T.....T........  24368
AP012544  21248  ...CG................C..T...........T.C............T........  21307
AP011548  30388  ...TG................C..T............G.......T.....T........  30329
FM179322  30388  ...TG................C..T............G.......T.....T........  30329
```

```
                     P40_casrham_for1                          P40_casrham_for2
Query     909     GGGTCAGTGCACCTGGTACGTCAAGTCAGTCGCTTCATGGGCTGGTAATGGCTGGGGCAA  968
HE970764  22500   ............................................................  22441
CP002618  22501   ............................................................  22442
CP002616  22501   ............................................................  22442
FM177140  22503   ............................................................  22444
CP006690  21342   ........................A.....T..AC........................  21283
CP001084  21851   ........................A.....T..AC........................  21792
CP005486  20022   ........................A.....T..AC........................  19963
CP005484  24367   ............A........T.....T..T...........A..C..............  24308
AP012544  21308   ......A..............T.....T..............A..C..............  21367
AP011548  30328   ............A........T.....T..T...........A..C..............  30269
FM179322  30328   ............A........T.....T..T...........A..C..............  30269

Query     969     TGGTGCCCAATGGGGTAGTTCCGCTGCAGCTGCTGGTTTCACAGTCAACCACACGCCAGC  1028
HE970764  22440   ............................................................  22381
CP002618  22441   ............................................................  22382
CP002616  22441   ............................................................  22382
FM177140  22443   ............................................................  22384
CP006690  21282   C..C..A........C.....A..C.....A.....C.....T..T..............  21223
CP001084  21791   C..C..A........C.....A..C.....A.....C.....T..T..............  21732
CP005486  19962   C..C..A........C.....A..C.....A.....C.....T..T..............  19903
CP005484  24307   C.....TG.......CGC............A..C..C.....G..T........C..G..  24248
AP012544  21368   C..C..TG.......CGC...............A..C.....G..T........A..G..  21427
AP011548  30268   C.....TG.......CGC............A..C..C.....G..T........C..G..  30209
FM179322  30268   C.....TG.......CGC............A..C..C.....G..T........C..G..  30209

Query     1029    AGCCGGTTCAATCATCGTCTTCGCCGCTGGTCAATCTGTTGGCGGTCAATGGACAGCCGA  1088
HE970764  22380   ............................................................  22321
CP002618  22381   ............................................................  22322
CP002616  22381   ............................................................  22322
FM177140  22383   ............................................................  22324
CP006690  21222   ......C.............................C.......................  21163
CP001084  21731   ......C.............................C.......................  21672
CP005486  19902   ......C.............................C.......................  19843
CP005484  24247   ...A.....C..............T................................T..  24188
AP012544  21428   ......C........T........T.....C.....A........C........T..T..  21487
AP011548  30208   ...A.....C..............T....................C...........T..  30149
FM179322  30208   ...A.....C..............T....................C...........T..  30149

Query     1089    TGGTTCATACGGTCACGTTGCTTACGTCCAATCTGTCTCTGGTGACAGTGTCACGATCAG  1148
HE970764  22320   ............................................................  22261
CP002618  22321   ............................................................  22262
CP002616  22321   ............................................................  22262
FM177140  22323   ............................................................  22264
CP006690  21162   .........T..............T...........T.......................  21103
CP001084  21671   .........T..............T...........T.......................  21612
CP005486  19842   .........T..............T...........T.......................  19783
CP005484  24187   ...C..T..T..............T..T.....C..T.....C.....C..T.......C  24128
AP012544  21488   ....CA...T.................T.....C..T..C..C.....C.....C..TTC  21547
AP011548  30148   ...C..T..T..............T..T........T.....C.....C..T.......C  30089
FM179322  30148   ...C..T..T..............T..T........T.....C.....C..T.......C  30089

Query     1149    CCAAGGCGGCATGGGCTTCAGCTCACCAACCGGCCCGAACACCCAAACCATCTCTGGTGC  1208
HE970764  22260   ............................................................  22201
CP002618  22261   ............................................................  22202
CP002616  22261   ............................................................  22202
FM177140  22263   ............................................................  22204
CP006690  21102   ............................................................  21043
CP001084  21611   ............................................................  21552
CP005486  19782   ............................................................  19723
CP005484  24127   T........T..........................T........G..............  24068
AP012544  21548   .............................................G...........C..  21607
AP011548  30088   T................................T..T........G..............  30029
FM179322  30088   T................................T..T........G..............  30029

Query     1209    AAGCAGTTACGTTTATATCCACCGGTAA  1236
HE970764  22200   ............................  22173
CP002618  22201   ............................  22174
CP002616  22201   ............................  22174
FM177140  22203   ............................  22176
CP006690  21042   ......C.....................  21015
CP001084  21551   ......C.....................  21524
```

```
CP005486  19722  ......C.....................  19695
CP005484  24067  C.....C........C............  24040
AP012544  21608  ......C...AC................  21635
AP011548  30028  C.....C........C............  30001
FM179322  30028  C.....C........C............  30001
```

Table S5.- Blast alignment of *L.casei/paracasei/rhamnosus* genes encoding P75 proteins for the selection of PCR primers

```
Database: Nucleotide collection (nt)
           36,527,618 sequences; 117,866,738,766 total letters
Query= gi|191636824|ref|NC_010999.1| Lactobacillus casei BL23 complete
genome, strain BL23

Length=3079196


                                                                  Score     E
Sequences producing significant alignments:                      (Bits)  Value

emb|HE970764.1|  Lactobacillus casei W56 complete genome           2743    0.0
gb|CP002618.1|  Lactobacillus casei BD-II, complete genome         2743    0.0
gb|CP002616.1|  Lactobacillus casei LC2W, complete genome          2743    0.0
emb|FM177140.1|  Lactobacillus casei BL23 complete genome, str...  2743    0.0
gb|CP006690.1|  Lactobacillus casei 12A, complete genome           2604    0.0
gb|CP001084.2|  Lactobacillus casei str. Zhang, complete genome    2379    0.0
gb|CP005486.1|  Lactobacillus casei LOCK919, complete genome       2379    0.0
gb|CP005484.1|  Lactobacillus rhamnosus LOCK900, complete genome   420     2e-116
dbj|AP011548.1|  Lactobacillus rhamnosus ATCC 53103 DNA, compl...  420     2e-116
emb|FM179322.1|  Lactobacillus rhamnosus GG whole genome seque...  420     2e-116
gb|CP014645.1|  Lactobacillus rhamnosus strain ASCC 290 genome     409     4e-113
emb|LT220504.1|  Lactobacillus rhamnosus strain BPL5 genome as...  409     4e-113
gb|CP005485.1|  Lactobacillus rhamnosus LOCK908, complete genome   409     4e-113
gb|CP003094.1|  Lactobacillus rhamnosus ATCC 8530, complete ge...  409     4e-113
emb|FM179323.1|  Lactobacillus rhamnosus Lc 705 whole genome s...  409     4e-113

ALIGNMENTS

Query     277055   ATGGTAGATGCAAAGAAAGTATTGTCAGTTACGGCAGGATTCGTTGGTGCTGCCGGTTTG  277114
HE970764  277026   ............................................................  277085
CP002618  274360   ............................................................  274419
CP002616  277061   ............................................................  277120
FM177140  277055   ............................................................  277114
CP006690  294753   ............................................................  294812
CP001084  294624   ............................................................  294683
CP005486  290686   ............................................................  290745
CP005484  295348   .........T.T.................A........C..................C..  295407
AP011548  324696   .........T.T.................A........C..................C..  324755
FM179322  325672   .........T.T.................A........C..................C..  325731
CP014645  102073   .........T.T.................A........C..................C..  102132
LT220504  1953631  .........T.T.................A........C..................C..  1953572
CP005485  315086   .........T.T.................A........C..................C..  315145
CP003094  307877   .........T.T.................A........C..................C..  307936
FM179323  307701   .........T.T.................A........C..................C..  307760

Query     277115   GCAACTTTAGCTACCGGCGCTAATACTGTTTCCGCTTCAACGGGGACAGTCAATTACAAA  277174
HE970764  277086   ............................................................  277145
CP002618  274420   ............................................................  274479
CP002616  277121   ............................................................  277180
FM177140  277115   ............................................................  277174
CP006690  294813   ............................................................  294872
CP001084  294684   ............................................................  294743
CP005486  290746   ............................................................  290805
CP005484  295408   ..GG.......A.....A..C.....C.....T..A..G..A.....G....G.......  295467
AP011548  324756   ..GG.......A.....A..C.....C.....T..A..G..A.....G....G.......  324815
FM179322  325732   ..GG.......A.....A..C.....C.....T..A..G..A.....G....G.......  325791
CP014645  102133   ..GG.......A.....A..C.....C.....T..A..G..A.....G....G.......  102192
LT220504  1953571  ..GG.......A.....A..C.....C.....T..A..G..A.....G....G.......  1953512
CP005485  315146   ..GG.......A.....A..C.....C.....T..A..G..A.....G....G.......  315205
CP003094  307937   ..GG.......A.....A..C.....C.....T..A..G..A.....G....G.......  307996
```

```
FM179323  307761   ..GG.......A.....A..C.....C.....T..A..G..A.....G....G.......  307820

Query     277175   TCGGGTGCGACCACCGTATGGAATAGTCCATCATGGCATCAAGTAAAACGCTATGTGACT  277234
HE970764  277146   ............................................................  277205
CP002618  274480   ............................................................  274539
CP002616  277181   ............................................................  277240
FM177140  277175   ............................................................  277234
CP006690  294873   ............................................................  294932
CP001084  294744   ............................................................  294803
CP005486  290806   ............................................................  290865
CP005484  295468   ..C...................................C.....C........C......  295527
AP011548  324816   ..C...................................C.....C........C......  324875
FM179322  325792   ..C...................................C.....C........C......  325851
CP014645  102193   ..C...................................C.....C........C......  102252
LT220504  1953511  ..C...................................C.....C........C......  1953452
CP005485  315206   ..C...................................C.....C........C......  315265
CP003094  307997   ..C...................................C.....C........C......  308056
FM179323  307821   ..C...................................C.....C........C......  307880

Query     277235   TTTGGTGACAAAGTACAAGTTCTAGGTCAAAAAGTTGACCGAAACGGTGCTACTTGGTAT  277294
HE970764  277206   ............................................................  277265
CP002618  274540   ............................................................  274599
CP002616  277241   ............................................................  277300
FM177140  277235   ............................................................  277294
CP006690  294933   ............................................................  294992
CP001084  294804   ............................................................  294863
CP005486  290866   ............................................................  290925
CP005484  295528   .....C....CG..G..GC.AT.....A...CC.......A...T...............  295587
AP011548  324876   .....G....CG..G..GC.AT.G...A...CC.......A...T...............  324935
FM179322  325852   .....G....CG..G..GC.AT.G...A...CC.......A...T...............  325911
CP014645  102253   .....C....CG..G..GC.AT.....A...CC.......A...T...............  102312
LT220504  1953451  .....C....CG..G..GC.AT.....A...CC.......A...T...............  1953392
CP005485  315266   .....C....CG..G..GC.AT.....A...CC.......A...T...............  315325
CP003094  308057   .....C....CG..G..GC.AT.....A...CC.......A...T...............  308116
FM179323  307881   .....C....CG..G..GC.AT.....A...CC.......A...T...............  307940

Query     277295   AAAGTCGGCGACAATCAGTGGATCCCTCTCATT-TATTTGAATTTTGACGGCAAGACTGT  277353
HE970764  277266   .................................-..........................  277324
CP002618  274600   .................................-..........................  274658
CP002616  277301   .................................-..........................  277359
FM177140  277295   .................................-..........................  277353
CP006690  294993   ...........................GG....-..........................  295051
CP001084  294864   ...........................GG....-..........................  294922
CP005486  290926   ...........................GG....-..........................  290984
CP005484  295588   .....T.................T..GGA-...G.........G...CG..T..A..G.C  295646
AP011548  324936   .....T.................T..GGA-...G.........G...CG..T..A....C  324994
FM179322  325912   .....T.................T..GGA-...G.........G...CG..T..A....C  325970
CP014645  102313   .....T.................T..GGA-...G.........G...CG..T..A..G.C  102371
LT220504  1953391  .....T.................T..GGA-...G.........G...CG..T..A..G.C  1953333
CP005485  315326   .....T.................T..GGA-...G.........G...CG..T..A..G.C  315384
CP003094  308117   .....T.................T..GGA-...G.........G...CG..T..A..G.C  308175
FM179323  307941   .....T.................T..GGA-...G.........G...CG..T..A..G.C  307999

Query     277354   CACCGTTCAAGCACC-AGAACAAACTGCCAGTCAGGCACCGGTTAGCCAAGCGCCAGCTA  277412
HE970764  277325   ...............-............................................  277383
CP002618  274659   ...............-............................................  274717
CP002616  277360   ...............-............................................  277418
FM177140  277354   ...............-............................................  277412
CP006690  295052   ...............-............................................  295110
CP001084  294923   ...............-............................................  294981
CP005486  290985   ...............-............................................  291043
CP005484  295647   ...G...G..A....G..TT.GG-.A..A....AA.TG.T..C........A..G....  295705
AP011548  324995   ...G...G..A....G..TT.GG-.A..A....AA.TG.T..C........A..G....  325053
FM179322  325971   ...G...G..A....G..TT.GG-.A..A....AA.TG.T..C........A..G....  326029
CP014645  102372   ...G...G..A....G..TT.GG-.A..A....AA.TG.T..C........A..G....  102430
LT220504  1953332  ...G...G..A....G..TT.GG-.A..A....AA.TG.T..C........A..G....  1953274
CP005485  315385   ...G...G..A....G..TT.GG-.A..A....AA.TG.T..C........A..G....  315443
CP003094  308176   ...G...G..A....G..TT.GG-.A..A....AA.TG.T..C........A..G....  308234
FM179323  308000   ...G...G..A....G..TT.GG-.A..A....AA.TG.T..C........A..G....  308058

Query     277413   GCCAAGCACCTGCAAGTCAGGCACCTGCGAGTCAAGCAGC--C-GCAC-AG--CCTGATA  277466
HE970764  277384   ........................................--.-....-..--.......  277437
CP002618  274718   ........................................--.-....-..--.......  274771
```

```
CP002616  277419   ........................................--.-....-..--.......  277472
FM177140  277413   ........................................--.-....-..--.......  277466
CP006690  295111   ........................................--.-....-..--.......  295164
CP001084  294982   ........................................--.-....-..--.......  295035
CP005486  291044   ........................................--.-....-..--.......  291097
CP005484  295706   .T..G..G...A....C..A.....A..A.CC...A..C.TG.A....C.CAAA.C....  295765
AP011548  325054   .T..G..G...A....C..A.....A..A.CC...A..C.TG.A....C.CAAA.C....  325113
FM179322  326030   .T..G..G...A....C..A.....A..A.CC...A..C.TG.A....C.CAAA.C....  326089
CP014645  102431   .T..G..G...A....C..A.....A..A.CC...A..C.TG.A....C.CAAA.C....  102490
LT220504  1953273  .T..G..G...A....C..A.....A..A.CC...A..C.TG.A....C.CAAA.C....  1953214
CP005485  315444   .T..G..G...A....C..A.....A..A.CC...A..C.TG.A....C.CAAA.C....  315503
CP003094  308235   .T..G..G...A....C..A.....A..A.CC...A..C.TG.A....C.CAAA.C....  308294
FM179323  308059   .T..G..G...A....C..A.....A..A.CC...A..C.TG.A....C.CAAA.C....  308118

Query     277467   CCCAGACAGC-AA-ACATTCAGTTATATGTTAAAAACATTGGTTCTGCAGTCACAGTTTG  277524
HE970764  277438   ..........-..-..............................................  277495
CP002618  274772   ..........-..-..............................................  274829
CP002616  277473   ..........-..-..............................................  277530
FM177140  277467   ..........-..-..............................................  277524
CP006690  295165   ..........-..-..............................................  295222
CP001084  295036   ..........-..-C.......................................C.....  295093
CP005486  291098   ..........-..-C.......................................C.....  291155
CP005484  295766   ....A.....T..T...--...C.T.....C..G..T..C.....A.....T..C..A..  295823
AP011548  325114   ....A.....T..T...--...C.T.....C..G..T..C.....A.....T..C..A..  325171
FM179322  326090   ....A.....T..T...--...C.T.....C..G..T..C.....A.....T..C..A..  326147
CP014645  102491   ....A.....T..T...--...C.T.....C..G..T..C.....A.....T..C..A..  102548
LT220504  1953213  ....A.....T..T...--...C.T.....C..G..T..C.....A.....T..C..A..  1953156
CP005485  315504   ....A.....T..T...--...C.T.....C..G..T..C.....A.....T..C..A..  315561
CP003094  308295   ....A.....T..T...--...C.T.....C..G..T..C.....A.....T..C..A..  308352
FM179323  308119   ....A.....T..T...--...C.T.....C..G..T..C.....A.....T..C..A..  308176

Query     277525   GGCAACACCAGCGTACAGTCAGGCAACTGGCCAGTATCTGGAAGGCAACCAGACGTTGAC  277584
HE970764  277496   ............................................................  277555
CP002618  274830   ............................................................  274889
CP002616  277531   ............................................................  277590
FM177140  277525   ............................................................  277584
CP006690  295223   ............................................................  295282
CP001084  295094   ............................................................  295153
CP005486  291156   ............................................................  291215
CP005484  295824   .A....C..G.....T.C...T..G..A..T..A.....A.......G......AC....  295883
AP011548  325172   .A....C..G.....T.C...T..G..A..T..A.....A.......G......AC....  325231
FM179322  326148   .A....C..G.....T.C...T..G..A..T..A.....A.......G......AC....  326207
CP014645  102549   .A....C..G.....T.C...T..G..A..T..A.....A.......G......AC....  102608
LT220504  1953155  .A....C..G.....T.C...T..G..A..T..A.....A.......G......AC....  1953096
CP005485  315562   .A....C..G.....T.C...T..G..A..T..A.....A.......G......AC....  315621
CP003094  308353   .A....C..G.....T.C...T..G..A..T..A.....A.......G......AC....  308412
FM179323  308177   .A....C..G.....T.C...T..G..A..T..A.....A.......G......AC....  308236

Query     277585   GGCGGTTGCCCAGTTGCAAGCCAATGGCGAAACATGGTATCGGTTAGCAAATGGCGGTTA  277644
HE970764  277556   ............................................................  277615
CP002618  274890   ............................................................  274949
CP002616  277591   ............................................................  277650
FM177140  277585   ............................................................  277644
CP006690  295283   ............................................................  295342
CP001084  295154   ............................................................  295213
CP005486  291216   ............................................................  291275
CP005484  295884   ...T.........CA......A..C..................C.T..C..C........  295943
AP011548  325232   ...T.........CA......A.....T...............C.T..C..C........  325291
FM179322  326208   ...T.........CA......A.....T...............C.T..C..C........  326267
CP014645  102609   ...T.........CA......A.....T.....G.........C.T..C..C........  102668
LT220504  1953095  ...T.........CA......A.....T.....G.........C.T..C..C........  1953036
CP005485  315622   ...T.........CA......A.....T.....G.........C.T..C..C........  315681
CP003094  308413   ...T.........CA......A.....T.....G.........C.T..C..C........  308472
FM179323  308237   ...T.........CA......A.....T.....G.........C.T..C..C........  308296

Query     277645   CGTGCCAGAACGTTTTGCTAGCACAACACCTGCACCAGCACCGCAGTCATCTGCTGCAAC  277704
HE970764  277616   ............................................................  277675
CP002618  274950   ............................................................  275009
CP002616  277651   ............................................................  277710
FM177140  277645   ............................................................  277704
CP006690  295343   ............................................................  295402
CP001084  295214   ............................................................  295273
CP005486  291276   ............................................................  291335
```

```
CP005484  295944   T..T..T.C...G....T............                                295973
AP011548  325292   T..T..T.C...G....T............                                325321
FM179322  326268   T..T..T.C...G....T............                                326297
CP014645  102669   T..T..T.C...G....T.........                                   102695
LT220504  1953035  T..T..T.C...G....T.........G..                                1953006
CP005485  315682   T..T..T.C...G....T.........                                   315708
CP003094  308473   T..T..T.C...G....T.........                                   308499
FM179323  308297   T..T..T.C...G....T.........                                   308323

Query     277705   GAGTGTTGCGGCACCGACTGCGCCTGTATCTGATGCAACGGCTTCGAACGCAGCTGCTTC  277764
HE970764  277676   ............................................................  277735
CP002618  275010   ............................................................  275069
CP002616  277711   ............................................................  277770
FM177140  277705   ............................................................  277764
CP006690  295403   ............................................................  295462
CP001084  295274   .......................................................-----  295328
CP005486  291336   .......................................................-----  291390

Query     277765   AAATGCCGCCGCTTCGGATGTTGCTGTCTCCAGTGCTGCAGCGTCTAGTGCCGCGGCTTC  277824
HE970764  277736   ............................................................  277795
CP002618  275070   ............................................................  275129
CP002616  277771   ............................................................  277830
FM177140  277765   ............................................................  277824
CP006690  295463   ............................................................  295522
CP001084  295329   ----------..........C.....C.........C........C.....T..A.....  295378
CP005486  291391   ----------..........C.....C.........C........C.....T..A.....  291440

Query     277825   ATTAGCTGCCGCTTCAGAAGCTGCTG---CGGTTGCGAATGCTTCTAGCGCTGCTGCATC  277881
HE970764  277796   ..........................---...............................  277852
CP002618  275130   ..........................---...............................  275186
CP002616  277831   ..........................---...............................  277887
FM177140  277825   ..........................---...............................  277881
CP006690  295523   ..........................---...............................  295579
CP001084  295379   ...G....T.................CTT.......A.....C.................  295438
CP005486  291441   ...G....T.................CTT.......A.....C.................  291500

Query     277882   -T-T-CTGC--T-GC-----C-GCTGTAG---CGAGTGCTGCCGCAGAATCCAGTGCTGC  277926
HE970764  277853   -.-.-....--.-..-----.-.......---............................  277897
CP002618  275187   -.-.-....--.-..-----.-.......---............................  275231
CP002616  277888   -.-.-....--.-..-----.-.......---............................  277932
FM177140  277882   -.-.-....--.-..-----.-.......---............................  277926
CP006690  295580   -.-.-....--.-..-----.-.......---..------------------........  295606
CP001084  295439   G.C.G....TG.A..GAGTG.T..C.C..AAT.C...A.G..T.................  295498
CP005486  291501   G.C.G....TG.A..GAGTG.T..C.C..AAT.C...A.G..T.................  291560

Query     277927   TGCAGAGTCCAGTGCTGCCGCATCGAAGGCTGCTGCTGATTCTAGCGCTGCAGCTGTGCA  277986
HE970764  277898   ............................................................  277957
CP002618  275232   ............................................................  275291
CP002616  277933   ............................................................  277992
FM177140  277927   ............................................................  277986
CP006690  295607   ............................................................  295666
CP001084  295499   ......A...........T.....T...................................  295558
CP005486  291561   ......A...........T.....T...................................  291620

Query     277987   GACTACGACGCCTGAGTCCAGTGCTGCGCCTGCAACGACACAGGTTGATGCAACTCAGGA  278046
HE970764  277958   ............................................................  278017
CP002618  275292   ............................................................  275351
CP002616  277993   ............................................................  278052
FM177140  277987   ............................................................  278046
CP006690  295667   ............................................................  295726
CP001084  295559   ............................................................  295618
CP005486  291621   ............................................................  291680

Query     278047   ACAGCAACAGCAGGCAGAACCAAGCAATACGGTCAATACCGAGGAAACGACAAATAACGC  278106
HE970764  278018   ............................................................  278077
CP002618  275352   ............................................................  275411
CP002616  278053   ............................................................  278112
FM177140  278047   ............................................................  278106
CP006690  295727   ............................................................  295786
CP001084  295619   ..........................T..........G......................  295678
CP005486  291681   ..........................T..........G......................  291740

Query     278107   GACGCCGACTCCTGCACCAACGCCGACGCCTGCACCAACCCCAGCCCCAGCTCCTGTTAC  278166
```

```
HE970764  278078   ............................................................  278137
CP002618  275412   ............................................................  275471
CP002616  278113   ............................................................  278172
FM177140  278107   ............................................................  278166
CP006690  295787   ............................................................  295846
CP001084  295679   ..............................................------........  295732
CP005486  291741   ..............................................------........  291794

Query     278167   GCCGTCACGTCAGGCAAAAATTCAGGCGGTTATCTCAATTGCTGAACAGCAAATTGGGAA  278226
HE970764  278138   ............................................................  278197
CP002618  275472   ............................................................  275531
CP002616  278173   ............................................................  278232
FM177140  278167   ............................................................  278226
CP006690  295847   ...................G........................................  295906
CP001084  295733   ............................................................  295792
CP005486  291795   ............................................................  291854

Query     278227   ACCTTATGTATGGGGTGGCAAGGGTCCTAACAGCTTTGACTGCTCAGGCTTGATGTATTA  278286
HE970764  278198   ............................................................  278257
CP002618  275532   ............................................................  275591
CP002616  278233   ............................................................  278292
FM177140  278227   ............................................................  278286
CP006690  295907   ............................................................  295966
CP001084  295793   ............................................................  295852
CP005486  291855   ............................................................  291914

Query     278287   TGCCTTTTTGAACGGCGCCGGTGTTAACATCGGTGGTTGGACAGTGCCACAAGAATCTTC  278346
HE970764  278258   ............................................................  278317
CP002618  275592   ............................................................  275651
CP002616  278293   ............................................................  278352
FM177140  278287   ............................................................  278346
CP006690  295967   ............................................................  296026
CP001084  295853   ............................................................  295912
CP005486  291915   ............................................................  291974

Query     278347   TGGTCAGCAAGTCTCACTGAGTGCACTTCAGCCTGGTGATTTGCTCTTCTGGGGCGGACA  278406
HE970764  278318   ............................................................  278377
CP002618  275652   ............................................................  275711
CP002616  278353   ............................................................  278412
FM177140  278347   ............................................................  278406
CP006690  296027   ............................................................  296086
CP001084  295913   ............................................................  295972
CP005486  291975   ............................................................  292034

Query     278407   TGGTAGCTCTTACCACGTAGCGCTCTACATTGGTGGCGGTACAATGATTCAGGCACCACA  278466
HE970764  278378   ............................................................  278437
CP002618  275712   ............................................................  275771
CP002616  278413   ............................................................  278472
FM177140  278407   ............................................................  278466
CP006690  296087   ............................................................  296146
CP001084  295973   ............................................................  296032
CP005486  292035   ............................................................  292094

Query     278467   ACCAGGTGAAAATGTGAAGTACACGGCATTAGCTTACTTCATGCCTGATTTTGCTGTTCG  278526
HE970764  278438   ............................................................  278497
CP002618  275772   ............................................................  275831
CP002616  278473   ............................................................  278532
FM177140  278467   ............................................................  278526
CP006690  296147   .....................T......................................  296206
CP001084  296033   .....................T......................................  296092
CP005486  292095   .....................T......................................  292154

Query     278527   TCCTTCACTATAA  278539
HE970764  278498   .............  278510
CP002618  275832   .............  275844
CP002616  278533   .............  278545
FM177140  278527   .............  278539
CP006690  296207   .............  296219
CP001084  296093   .............  296105
CP005486  292155   .............  292167
```

Aramini, J.M., Rossi, P., Huang, Y.J., Zhao, L., Jiang, M., Maglaqui, M., Xiao, R., Locke, J., Nair, R., Rost, B., Acton, T.B., Inouye, M., and Montelione, G.T. (2008). Solution NMR Structure of the NlpC/P60 Domain of Lipoprotein Spr from Escherichia coli: Structural Evidence for a Novel Cysteine Peptidase Catalytic Triad. *Biochemistry* 47**,** 9715-9717.

Källberg, M., Wang, H., Wang, S., Peng, J., Wang, Z., Lu, H., and Xu, J. (2012). Template-based protein structure modeling using the RaptorX web server. *Nature Protocols* 7**,** 1511.

Kankainen, M., Paulin, L., Tynkkynen, S., Von Ossowski, I., Reunanen, J., Partanen, P., Satokari, R., Vesterlund, S., Hendrickx, A.P.A., Lebeer, S., De Keersmaecker, S.C.J., Vanderleyden, J., Hämäläinen, T., Laukkanen, S., Salovuori, N., Ritari, J., Alatalo, E., Korpela, R., Mattila-Sandholm, T., Lassig, A., Hatakka, K., Kinnunen, K.T., Karjalainen, H., Saxelin, M., Laakso, K., Surakka, A., Palva, A., Salusjärvi, T., Auvinen, P., and De Vos, W.M. (2009). Comparative genomic analysis of Lactobacillus rhamnosus GG reveals pili containing a human- mucus binding protein. *Proceedings of the National Academy of Sciences* 106**,** 17193-17198.

Linares, D.M., Kok, J., and Poolman, B. (2010). Genome Sequences of Lactococcus lactis MG1363 (Revised) and NZ9000 and Comparative Physiological Studies. *Journal of Bacteriology* 192**,** 5806-5812.

Makarova, K., Slesarev, A., Wolf, Y., Sorokin, A., Mirkin, B., Koonin, E., Pavlov, A., Pavlova, N., Karamychev, V., Polouchine, N., Shakhova, V., Grigoriev, I., Lou, Y., Rohksar, D., Lucas, S., Huang, K., Goodstein, D.M., Hawkins, T., Plengvidhya, V., Welker, D., Hughes, J., Goh, Y., Benson, A., Baldwin, K., Lee, J.-H., Díaz-Muñiz, I., Dosti, B., Smeianov, V., Wechter, W., Barabote, R., Lorca, G., Altermann, E., Barrangou, R., Ganesan, B., Xie, Y., Rawsthorne, H., Tamir, D., Parker, C., Breidt, F., Broadbent, J., Hutkins, R., O'sullivan, D., Steele, J., Unlu, G., Saier, M., Klaenhammer, T., Richardson, P., Kozyavkin, S., Weimer, B., and Mills, D. (2006). Comparative genomics of the lactic acid bacteria. *Proceedings of the National Academy of Sciences* 103**,** 15611-15616.

Maze, A., Boel, G., Zuniga, M., Bourand, A., Loux, V., Yebra, M.J., Monedero, V., Correia, K., Jacques, N., Beaufils, S., Poncet, S., Joyet, P., Milohanic, E., Casaregola, S., Auffray, Y., Perez-Martinez, G., Gibrat, J.F., Zagorec, M., Francke, C., Hartke, A., and Deutscher, J.

(2010). Complete Genome Sequence of the Probiotic Lactobacillus casei Strain BL23. *The Journal of Bacteriology* 192**,** 2647-2648.

Siezen, R.J., Francke, C., Renckens, B., Boekhorst, J., Wels, M., Kleerebezem, M., and Van Hijum, S.a.F.T. (2012). Complete Resequencing and Reannotation of the Lactobacillus plantarum WCFS1 Genome. *Journal of Bacteriology* 194**,** 195-196.

Van De Guchte, M., Penaud, S., Grimaldi, C., Barbe, V., Bryson, K., Nicolas, P., Robert, C., Oztas, S., Mangenot, S., Couloux, A., Loux, V., Dervyn, R., Bossy, R., Bolotin, A., Batto, J.-M., Walunas, T., Gibrat, J.-F., Bessières, P., Weissenbach, J., Ehrlich, S.D., and Maguin, E. (2006). The complete genome sequence of Lactobacillus bulgaricus reveals extensive and ongoing reductive evolution. *Proceedings of the National Academy of Sciences* 103**,** 9274-9279.

Wang, S., Li, W., Liu, S., and Xu, J. (2016). RaptorX-Property: a web server for protein structure property prediction. *Nucleic Acids Research* 44**,** W430-W435.

Waterhouse, A., Bertoni, M., Bienert, S., Studer, G., Tauriello, G., Gumienny, R., Heer, F.T., De Beer, T.a.P., Rempfer, C., Bordoli, L., Lepore, R., and Schwede, T. (2018). SWISS-MODEL: homology modelling of protein structures and complexes. *Nucleic Acids Research* 46**,** W296-W303.

Wegmann, U., O'connell-Motherway, M., Zomer, A., Buist, G., Shearman, C., Canchaya, C., Ventura, M., Goesmann, A., Gasson, M.J., Kuipers, O.P., Van Sinderen, D., and Kok, J. (2007). Complete Genome Sequence of the Prototype Lactic Acid Bacterium Lactococcus lactis subsp. cremoris MG1363. *Journal of Bacteriology* 189**,** 3256-3270.

Yan, F., Cao, H., Cover, T.L., Whitehead, R., Washington, M.K., and Polk, D.B. (2007). Soluble Proteins Produced by Probiotic Bacteria Regulate Intestinal Epithelial Cell Survival and Growth. *Gastroenterology* 132**,** 562-575.
